# Targeting CXCL8 signaling sensitizes HNSCC to anlotinib by reducing tumor-associated macrophage-derived CLU

**DOI:** 10.1186/s13046-025-03298-7

**Published:** 2025-02-05

**Authors:** Xin Hu, Yikang Ji, Mi Zhang, Zhihui Li, Xinhua Pan, Zhen Zhang, Xu Wang

**Affiliations:** 1 Department of Oral and Maxillofacial-Head and Neck Oncology, Shanghai Ninth People’s Hospital, Shanghai Jiao Tong University School of Medicine, Shanghai, 200011 China; 2 National Clinical Research Center for Oral Disease, Shanghai, 200011 China; 3 Shanghai Key Laboratory of Stomatology & Shanghai Research Institute of Stomatology, Shanghai, 200011 China; 4 Digital Diagnosis and Treatment Innovation Center for Cancer, Institute of Translational Medicine, Shanghai Jiao Tong University, Shanghai, 200240 China

**Keywords:** CXCL8, Oxidative stress, CLU, Nutrition starvation, HNSCC

## Abstract

**Background:**

Although nutrition-starvation therapy for malignancies such as HNSCC is highly desirable, the clinical outcomes remain disappointing. Understanding the spatial heterogeneity of glucose deficiency can reveal the molecular mechanisms regulating cancer metabolism and identify therapeutic targets to improve effective nutrient-starvation therapies.

**Methods:**

Multiple omics data from RNA-seq, proteomics and spatial transcriptome analyses of HNSCC samples were integrated to analyze the spatial heterogeneity of glucose deficiency. In vivo and in vitro CXCL8 and CLU expression levels in tumor cells were determined using qPCR, immunohistochemistry and ELISA. The ability of CLU from TAMs to respond to tumor-derived CXCL8 was assessed using RNA sequencing, siRNA silencing, immunofluorescence and CCK-8 assays. A mouse subcutaneous xenograft model was used to assess the outcomes of nutrition-starvation therapy combined with blockade of CXCL8 signaling.

**Results:**

A set of genes that was significantly upregulated in HNSCC under conditions of glucose deficiency was identified using integrating multiple omics data analyses. The upregulated gene set was used to determine the glucose-deficient area according to transcriptome data of HNSCC, and *CXCL8* was one of the most highly upregulated genes. The levels of both *CXCL8* mRNA and its protein IL-8 in cancer cells under conditions of glucose deficiency were increased in an NF-κB-dependent manner. Supplementary IL-8 stimulated TAMs to synthesize CLU, and CLU counteracted oxidative stress in HNSCC cells under conditions of glucose deficiency. Moreover, pharmacological blockade of CXCL8 signaling (reparixin) sensitized HNSCC cells to nutrient-starvation therapy (anlotinib) in two xenograft models.

**Conclusion:**

Our results provide novel evidence of a feedback loop between cancer cells and TAMs in glucose-deficient regions. HNSCC-derived CXCL8 favors endogenous antioxidative processes and confers therapeutic resistance to nutrient-starvation therapies in HNSCC.

**Graphical Abstract:**

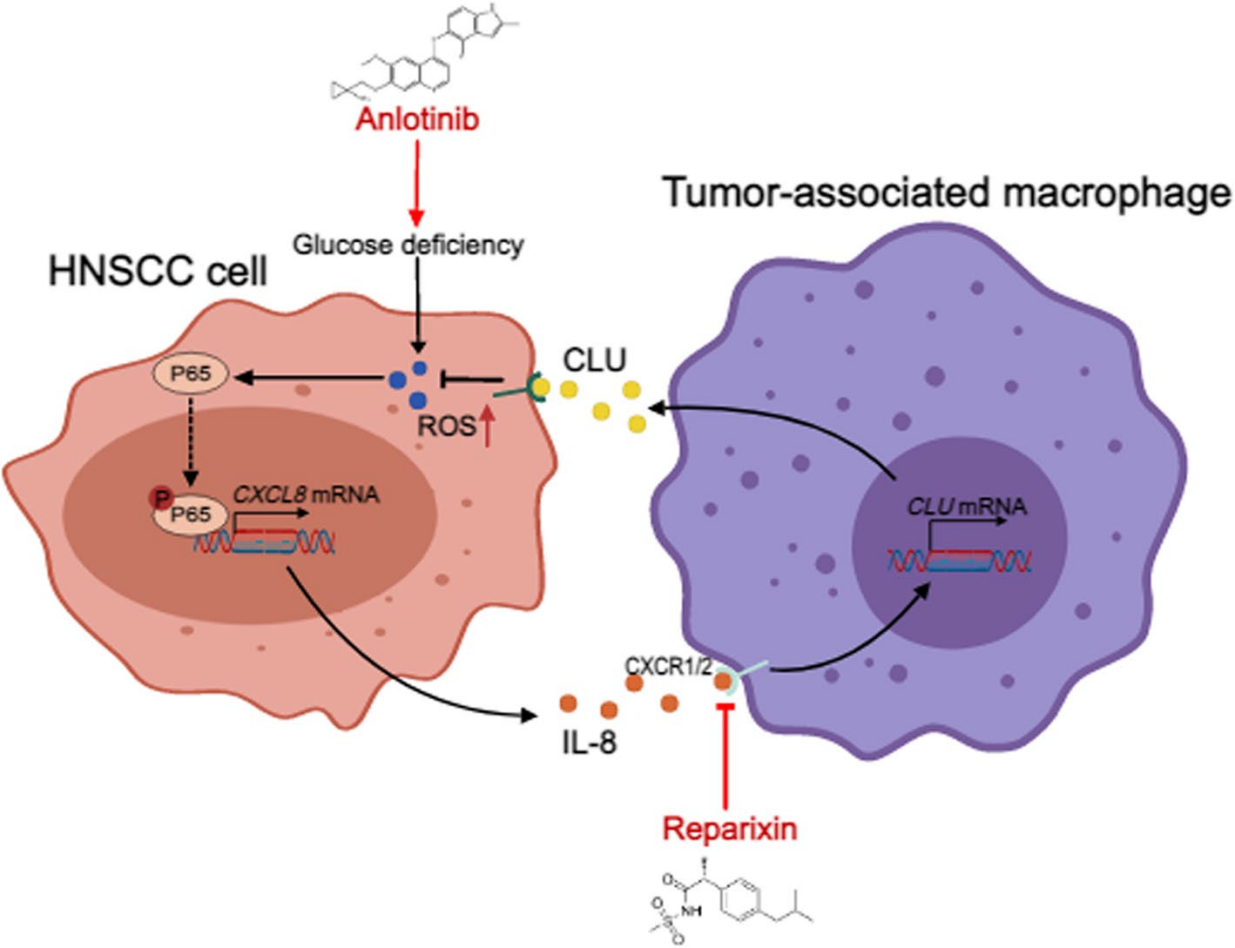

**Supplementary Information:**

The online version contains supplementary material available at 10.1186/s13046-025-03298-7.

## Introduction

Glucose serves as the primary source of cellular energy, generating ATP through glycolysis, the citric acid cycle, and oxidative phosphorylation [1]. However, in malignancies such as head and neck squamous cell carcinoma (HNSCC), the demand for glucose significantly increases because of the rapid proliferation and metabolic reprogramming of cancer cells [2]. To target this feature of tumor cells, nutrition-starvation therapies have been developed to reduce the supply of glucose and other nutrients to cancer cells. For example, the tyrosine kinase inhibitor anlotinib inhibits tumor angiogenesis by targeting VEGFR, FGFR, and PDGFR and has been used in the clinical treatment of various malignant tumors [3, 4]. However, the outcomes of nutrient-starvation therapy in HNSCC patients remain disappointing [5]. Therefore, it is necessary to further explore the molecular mechanisms regulating tumor dissemination and therapeutic resistance to develop effective cancer therapies.

Cancer cells can evade the oxidative stress caused by nutrient-starvation therapy through various mechanisms, leading to drug resistance [6]. For example, cancer cells can self-secrete cytokines to reduce oxidative stress [7]. Oxidative stress plays a pivotal role in cancer metabolism, particularly under nutrient-deprived conditions like glucose deficiency. Cancer cells often face increased reactive oxygen species (ROS) levels due to metabolic reprogramming and disrupted redox balance. To survive, they activate antioxidant mechanisms, such as upregulating glutathione synthesis or relying on tumor-associated macrophages (TAMs) for antioxidant support. This adaptive response not only promotes cell survival but also contributes to therapeutic resistance, making oxidative stress a key target for overcoming cancer treatment challenges. TAMs can also secrete some antioxidants to protect tumor cells from excessive oxidative stress damage [8]. The distribution and utilization of glucose exhibit notable spatial heterogeneity within HNSCC tissues. This heterogeneity stems from various factors, including irregular angiogenesis, a complicated molecular microenvironment, and metabolic competition among different cells [9]. Analysis of the spatial transcriptome provides an effective technical method to analyze gene expression differences [10]; however, at present, methods for identifying sugar-deficient regions within tumors are lacking. By integrating multiomics data from cancer cells under conditions of glucose deficiency, researchers can reveal a new approach to illustrate how cancer cells tolerate the glucose-deficient microenvironment and discover novel therapeutic targets.

The CXCL8 signaling pathway, centered on the chemokine CXCL8 (also known as interleukin-8, IL-8) and its receptors CXCR1 and CXCR2, is crucial in neutrophil chemotaxis and inflammation [11]. Cancer cells often secrete IL-8 to attract immune cells, creating an inflammatory microenvironment that promotes tumor growth and metastasis. The nuclear factor kappa B (NF-κB) complex plays a pivotal role in regulating inflammatory reactions. NF-κB/p65 protein phosphorylated at the Ser536 residue binds the promoter of the *CXCL8* gene and enhances CXCL8 expression in triple-negative breast cancers [12]. Reparixin is a noncompetitive allosteric inhibitor of the CXCL1/2 chemokine receptor that reduces the inflammatory response. It has been shown in phase 3 clinical trials to prevent ischemia‒reperfusion injury and inflammation [13] and is in a clinical trial for the treatment of triple-negative breast cancer [14, 15]. However, it is important to note that its clinical applications remain in the early stages. Further studies, including clinical trials, are needed to fully understand its efficacy and safety to overcome the resistance that cancers often develop to single agent treatments, including nutrient-starvation therapy.

Clusterin (CLU), also known as apolipoprotein J, is a sulfated chaperonic glycoprotein expressed in all tissues and human fluids and has been reported as a potential biomarker of OSCC [16]. Abnormal CLU expression is associated with cancer development and progression. CLU plays a vital role in many biological processes, such as apoptosis and the cell cycle [17, 18]. For example, secretory CLU promotes oral cancer cell survival by inhibiting apoptosis through the activation of autophagy [19]. CLU can protect cells from reactive oxygen species-mediated cell death [20]. However, the functional implications of CLU in nutrient-starvation therapy lack detailed mechanistic insights.

In this study, we report that in HNSCC, CXCL8 expression is activated via NF-κB/p65 protein phosphorylation at the Ser536 site by ROS induced under conditions of glucose deficiency, whereas CXCL8 increases CLU expression in TAMs to facilitate antioxidative stress in cancer cells. Pharmalogical blockade of CXCL8 signaling synergistically inhibited HNSCC growth with nutrient-starvation therapy. Our findings reveal the molecular mechanism by which HNSCC tolerates a glucose-deficient microenvironment by educating TAMs, suggesting that targeting CXCL8 represents a promising therapeutic strategy to increase the efficacy of nutrient starvation therapies for HNSCC.

## Methods

### Animals

All BALB/c nude and C3H/HeJ mice (6–7 weeks old) were raised under pathogen-free conditions in the animal care facilities of the Ninth People’s Hospital, Shanghai Jiao Tong University School of Medicine. All animals lived at room temperature (22–25 °C) and had food and water with free access under a 12-h light/dark cycle. All mice used in experiments were acclimatized in the breeding room for a week before treatment. All animal studies were performed in accordance with the Guide for Care and Use of Laboratory Animals (The Ministry of Science and Technology of China, 2006) and the appropriate ethical regulations of the hospital. All experimental procedures were approved by the Laboratory Animal Care and Use Committee of the hospital.

### Cell culture

The human HNSCC cell lines HN6, HN30, CAL27 were all cultured in DMEM/F12 medium (BasalMedia, China) supplemented with 10% fetal bovine serum (FBS; Cellmax, China) and 1% penicillin–streptomycin (PS; NCM Biotech, China). The human oral keratinocytes (HOK), murine squamous cell carcinoma cell line SCC7 and abelson murine leukemia virus-induced tumor cell line RAW264.7 were maintained in high glucose DMEM medium (BasalMedia, China) supplemented with 10% FBS and 1% PS. The human acute monocytic leukemia cells (THP-1) were maintained in RPMI 1640 medium (BasalMedia, China) supplemented with 10% FBS, 1% PS and 50 μM 2-mercaptoethanol (Macklin, China). All cells were incubated in a humidified atmosphere with 5% CO_2_ at 37 °C.

### Primary culture of mouse BMDMs

The femurs and tibias of the hind limbs of 8-week-old male mice were isolated, and the bone marrow cells were removed with a sterile syringe and collected in 50 ml of sterile PBS, filtered through a 70 μm cell strainer, and centrifuged for 5 min at 450 g. Red blood cells were lysed with red blood cell lysis buffer, the sediment was resuspended and cultured in RPMI 1640 (BasalMedia, China) supplemented with 10% FBS, 1% PS and 50 μM 2-mercaptoethanol (Macklin, China) in the presence of 20 ng/ml macrophage colony-stimulating factor (M-CSF; PeproTech, US) for 5–7 days. Cells were then harvested for further experiments.

### Culture of human M2-like macrophages

To obtain THP-1-derived M2-like macrophages, THP-1 cells were maintained in RPMI 1640 (BasalMedia, China) supplemented with 10% FBS, 1% PS and 50 μM 2-mercaptoethanol (Macklin, China) and treated with 100 ng/ml Phorbol 12-myristate 13-acetate (PMA; MCE, China) for 48 h, and then cultured with 20 ng/ml IL-4 (Novoprotein, China) and 20 ng/ml IL-13 (Novoprotein, China) for another 48 h. Cells were then harvested for further experiments.

### Macrophages derived conditional media (CM)

RAW264.7, murine BMDMs and THP-1-derived M2-like macrophages were planted on 6-well plates for 24 h and then treated with 100 ng/ml recombinant human IL-8 protein (SinoBiological, China) or 100 ng/ml recombinant mouse Cxcl15 protein (Novoprotein, China) for another 24 h in glucose-free DMEM (BasalMedia, China) with 1 mM D-glucose (MCE, China). The conditional media was then collected, centrifuged at 450 g for 5 min, filtered through a 0.22 mm filter, and 1:1.5 diluted by fresh artificial low-glucose medium.

### Cell viability assays

HN6, SCC7 cells were seeded in 96-well plates (6,000 cells per well). Cancer cells were then cultured with indicated CM for 48 h and the CCK-8 assay was used to determine cell viability. CCK-8 reagent (NCM Biotech, China) was diluted 10 times with FBS-free medium and replaced with the original medium (100 μL per well). After incubating at 37 °C for 2 h, the OD value of each well was measured at 450 nm using a microplate reader (SpectraMax i3, Molecular Devices, USA).

### ROS detection

To determine the intracellular ROS levels in HN6 and SCC7 cell line under glucose-deprivation or other treatment, HN6 and SCC7 cell line were seeded in 35 mm cell culture dishes with the optical characteristics of coverslips with 0.5 mL high-glucose DMEM medium or seeded in 96-well plates (6,000 cells per well) with 100 μL medium. All plates or dishes were treated with conditional media or other media 24 h later. Intracellular ROS levels in cell lines were analyzed by a ROS assay kit with DCFH-DA probe (Beyotime, China) according to the manufacturer’s instructions. The mean fluorescence density was analyzed via using software ImageJ.

### Quantitative real-time PCR (qPCR)

Total RNA was extracted with RNA-Quick Purification Kit (Yishan, China) according to the manufacturer’s protocol, and cDNA was synthesized using the PrimerScript RT reagent Kit (Takara, Japan). The qPCR reactions were performed using Hieff UNICON® qPCR SYBR Green Master Mix (Yeasen, China). The amplified PCR products were quantified and normalized using β-Actin as a reference gene. The concentration of the primers was 0.2 μM and the sequences or consumables used in qPCR were listed in Supplementary Materials and Table.

### Western blotting assay

For western blotting assay, cells were lysed with RIPA lysis buffer (NCM Biotech, China) and subjected to protein quantification using the BCA Protein Assay Kit (Thermo Fisher Scientific, USA). The protein samples were added with a quarter volume 5 × loading buffer (NCM Biotech, China) and incubated at 105 °C for 10 min. Then the protein samples were separated by polyacrylamide gels (Genescrpit, China) and transferred to 0.22 μm polyvinylidene fluoride (PVDF) membranes (Merck Millipore, USA). The blots were blocked with 5% skimmed milk in TBST for 2 h at room temperature. After washed with TBST for 15 min, the blots were incubated with specific primary antibodies overnight at 4 °C. Afterward, the membranes were probed with HRP-conjugated secondary antibody (Beyotime, China; A0208, 1:1000) and visualized by NcmECL Ultra Enhanced Chemiluminescent reagent (NCM Biotech, China) and Amersham Imager 600 system (General Electric Company, USA). β-Actin was used as a loading control. The primary antibodies used in this study are anti-NF-kB p65/RelA (Abclonal, China; A19653, 1:5000), anti-Phospho-NF-kB p65/RelA-S536 (Abclonal, China; AP0124, 1:2000) and anti-β-actin (Abclonal, China; AC026, 1:50000).

### Enzyme-linked immunosorbent assay (ELISA)

To detect the active form of IL-8 or Cxcl15 content secreted from OSCC cell lines under glucose-deficiency, cancer cells were seeded in 12-well plates with 500 μL glucose-free DMEM medium for 18 h. To detect the active form of CLU or Clu content secreted from macrophages activated by IL-8 or Cxcl15, macrophages were planted in 12-well plates with 500 μL high-glucose DMEM medium with or without recombinant IL-8 and Cxcl15 protein for 24 h. Cell culture supernatant was collected and centrifuged at 1000 g for 20 min. Human IL-8, CLU and mouse Cxcl15, Clu content were respectively analyzed by the human IL-8, CLU and mouse Cxcl15, Clu ELISA kits according to the manufacturer’s instructions (Hengyuan Biotechnology, China). The optical density (OD value) of each well was measured at 450 nm using a plate reader (SpectraMax i3, Molecular Devices, USA).

### Extracellular and intracellular glucose consumption assay

HOK, HN6 and SCC7 cells were cultured until 50% of confluency and then changed to a fresh high glucose DMEM medium. After 24 h, the supernatants and the remained cells were collected for measurement of glucose concentrations. Glucose levels were determined using a Glucose (GOD-POD) Assay Kit (Yeasen, China) and glucose content was normalized to cellular protein concentrations. Of note, extracellular glucose consumption was the difference in glucose concentration from the collected culture medium between experimental conditions. All operations follow the kit instructions.

### siRNA interference

RAW264.7 cell line was seeded in 6-well plates with high-glucose DMEM medium. When reached approximately 30% density, RNAiMAX was used as the transfection reagent according to the manufacturer’s instructions (Thermo Fisher Scientific, USA). RAW264.7 cells were incubated with 50 nM siRNA for 24 h, and then the medium was replaced with fresh 10% FBS DMEM medium containing 100 ng/ml recombinant mouse Cxcl15 protein. The siRNAs used in this study were designed and synthesized by GenePharma, China. The siRNA sequences are listed in the Supplementary Table.

### Proteomics

Proteomics examination was performed with OEbiotech (Shanghai, China) as previously described [7]. In brief, after HN6 cells grow at 90% confluency, replace with either glucose-free medium or complete medium for 8 h, Repeat the washing step with pre-chilled PBS for three times Use a clean cell scraper to scrape the cells to one side of the dish. Transfer the lysed solution to a pre-chilled centrifuge tube. Centrifuge and discard the supernatant. Flash freeze with liquid nitrogen and store at -80 °C. Protein was extracted and digested. LC–MS/MS analysis was performed on a timsTOF Pro mass spectrometry (Bruker) that was coupled to Nanoelute (Bruker). The MS raw data for each sample were combined and searched using the MaxQuant 1.6.14 software for identification and quantitation analysis. |Fold change|> 1.5 and adjusted *P* value < 0.05 were considered the threshold. Schematic diagram of transcriptome and proteomics was generated with the online tool from biorender (https://app.biorender.com/user/signup). Nine-quadrant diagram and Heatmap of significantly changed genes were generated with the online tool from OE cloud (https://cloud.oebiotech.com/). GO enrichment analysis was performed with the online tool from metascape (http://metascape.org).

### RNA sequencing

RNA-seq analysis was performed with OEbiotech (Shanghai, China). In brief, total RNA was extracted using TRIzol reagent (Invitrogen) according to the manufacturer’s protocol for the indicated cell samples. Transcriptome sequencing of RNA was completed by OEbiotech. The differentially expressed genes (DEGs) between groups were screened using the limma package in R. |Fold change|> 1.5 and adjusted *P* value < 0.05 were considered the threshold.

### Mouse subcutaneous xenografts model

Male BALB/c nude mice and C3H/HeJ mice aged 6–8 weeks were selected in this experiment. 100 μL of cell suspension (500,000 SCC7 cells or 1,000,000 HN6 cells re-suspended in PBS) was subcutaneously injected into the right flank of nude mice after general anesthesia with 1% pentobarbital sodium. For macrophage depletion, mice were injected i.p. with 200 μl of liposomal clodronate (SunLipo NanoTech) on days 7 after tumor inoculation and then 100 μl every two weeks. The body weight of mice was recorded every other day. Tumor growth was monitored by measuring the tumor dimensions using calipers and the tumor volume was calculated using the following formula: V = (L × [W]^2^) × 0.5, where L is the longest diameter of the tumor and W is the shortest diameter of the tumor. On the 7th day, after confirming tumor establishment, mice were divided into four groups and performed the indicated treatment (*n* = 5 or 6 per group). Anlotinib was administered 3 mg/kg only one time at day 1 after grouping. Reparixin was injected intraperitoneally at 3 mg/kg every day. Once the tumor volume exceeded 2,000 mm^3^, euthanasia by CO_2_ inhalation was performed. Tumors were photographed, weighed, and then fixed with 4% paraformaldehyde for histological analysis.

### H&E staining and immunohistochemistry (IHC)

For H&E staining, paraffin-embedded 3 μm thick sections were deparaffinized and rehydrated. Then Hematoxylin and dehydration were used to counterstain the nucleus. Eosin was used to counterstain the cytoplasm, and slides were submerged into graded ethanol and xylene and covered with coverslips. For IHC, paraffin‐embedded 3 μm thick sections were deparaffinized, rehydrated, submerged into citric acid buffer for heat‐induced antigen retrieval, and immersed in 0.3% hydrogen peroxide to block endogenous peroxidase activity. After blocked with 3% goat serum albumin, the sections were incubated with primary antibodies at 4 ℃ overnight. Afterward, the sections were incubated with HRP-conjugated secondary antibody (Beyotime, China; A0208, 1:50) at room temperature for 1 h. Hematoxylin and dehydration were used to counterstain the nucleus. Then slides were submerged into graded ethanol and xylene and covered with coverslips. The pictures were scanned by panoramic slice scanner (Pannoramic MIDI/P250, 3DHISTECH, Hungary). The antibodies used in this experiment are anti-IL-8 (Proteintech, China; 27,095-1-AP, 1:100) and anti-KI67 (Proteintech, China; 27,309-1-AP, 1:2000). The mean density was analyzed via using software ImageJ.

### Immunofluorescence

For cell immunofluorescence assays, HN6 and SCC7 cell lines were cultured in confocal dishes under glucose-deficiency for 16 h, and then fixed with 4% paraformaldehyde. For tissue immunofluorescence assays, paraffin‐embedded 3 μm thick sections were deparaffinized, rehydrated, and submerged into citric acid buffer for heat‐induced antigen retrieval. After washed with TBST, the sections were penetrated with 0.5% Triton X-100 and immersed in 0.3% hydrogen peroxide to block endogenous peroxidase activity. Then blocked with 3% goat serum albumin, the sections were incubated with primary antibodies at 4 ℃ overnight. Then samples were stained with Alexa Fluor 647-conjugated secondary antibodies (Beyotime,China; A0468, 1:500) for 1 h at room temperature. Nuclei was visualized by 4′,6-diamidino-2-phenylindole (DAPI; Beyotime, China; 1:100) staining. Images were obtained with ZEISS LSM880 scanning confocal microscope (ZEISS, Germany). The primary antibodies used in this experiment are anti-CLU (Abclonal, China; A13479, 1:50), anti-F4/80 (Proteintech, China; 28,463-1-AP, 1:50). The mean fluorescence density was analyzed via using software ImageJ.

### Data collection

The pan-cancer 10X Visium spatial trancriptomic data were collected from published data set, the head and neck squamous cancer data was from GSE220978 and code GSE208253. The direct pathogenic factor of 4 patients in GSE220978 is oral submucous fibrosis caused by betel nuts chewing, the researchers did not check the HPV infection status. In GSE220978, all samples are HPV-negative. The clear cell renal carcinoma data was from GSE175540, the colorectal cancer data was from GSE225857, the cutaneous squamous cell carcinoma data was from GSE144239, the hepatocellular carcinoma data was from GSE238264, the non-small cell lung cancer data was from E-MTAB-13530 and ovarian cancer data was from GSE211956.

### Data preprocessing

Quality control of the 10X Visium data was performed with R (version 4.3.3) and Seurat [21] software (version 4.4.0). The spatial spots were evaluated with number of features and percentage of mitochondrial genes, low quality spots with less than 300 genes or more than 10% of mitochondrial genes were discarded. The data was integrated with merge function and batch effect was removed with Harmony, Then Normalization, SCTransform and dimensional reduction (PCA, UMAP and t-SNE) were performed with Seurat.

### Biological enrichment analysis

ClusterProfiler [22] was used for gene set enrichment analysis (GSEA) and gene set variation analysis (GSVA). The gene set terms were downloaded from gsea [23, 24] website. The geneset evaluations were added to the seurat object and visulized with Spatialfeatureplot function to show their distribution in the tissue slide.

### Single-cell data analysis

Seurat was used for the processing of single cell transcriptomics data. The UMI count matrix was preprocessed using the Seurat R package. Genes expressed in more than three cells were retained for further analysis, while cells with fewer than 200 genes or more than 10% mitochondrial content were filtered out. PCA was conducted based on the 2,000 most variable genes, and the first 20 principal components were used for t-SNE and UMAP dimensionality reduction. Both t-SNE and UMAP were performed using the default settings of the RunTSNE and RunUMAP functions in Seurat. Cell type annotation was conducted with SingleR [25] (v1.8.1) and CellMarker2.0 [26].

### Visium spots annotation and nearest neighbors’ analysis

Robust cell type decomposition (RCTD) [27] method was used for spot deconvolution of HNSCC visium spot, the pan-cancer visium spot annotation was perform with xCell [28] algorithm. The nearest 6 spots around the visium were considered for nearest neighbors, DBSCAN [29] (Density-Based Spatial Clustering of Applications with Noise) algorithm was applied for calculation of molecular neighbors and cell neighbors.

### Tumor boundary identification and spatial gradient analysis

R package Cottrazm [30] was used for recognition of malignant spots, non-malignant spots and boundary spot of the visium data followed the standard protocol. The cancer-like clusters were identified with BoundaryDefine function, the malignant spots, boundary spost and non-malignant spots were projected to spatial HE staining plot with BoundaryPlot function. Local spatial gradient inference (LGSI) [31] was adopted for computation of spatial gradient of gene expression or signaling pathway levels.

### Spatial cell–cell communications

The python package COMMOT [32] was used for inferring cell–cell interactions in spatial transcriptomics data. The 10X Visium data was firstly process with a standard procedure of scanpy [33], CellChat [34] database were adopted for cell–cell interactions as well as spatial signal stream analysis.

### Statistical analysis

All data were presented as means ± standard error of mean (SEM). The normal distribution and homogeneity of the data were tested by SPSS. Unless otherwise stated, unpaired or paired 2-tailed student’s t-test was used to test the significance of differences between the two groups. Two-way analysis of variance (ANOVA) was used to compare the effects of different factors on mouse tumor volume. Statistical analyses were conducted using GraphPad Prism and R Software. The exact sample size (n) for each experimental group/condition, was given as a discrete number and unit of measurement. *p* values less than 0.05 were considered statistically significant, the level of significance was set at *p* < 0.05 (*), *p* < 0.01 (**), *p* < 0.01 (***) or *p* < 0.001 (****).

## Results

### Integrated multiomics analysis identifies the spatial heterogeneity of glucose deficiency in HNSCC

**Fig. 1:**
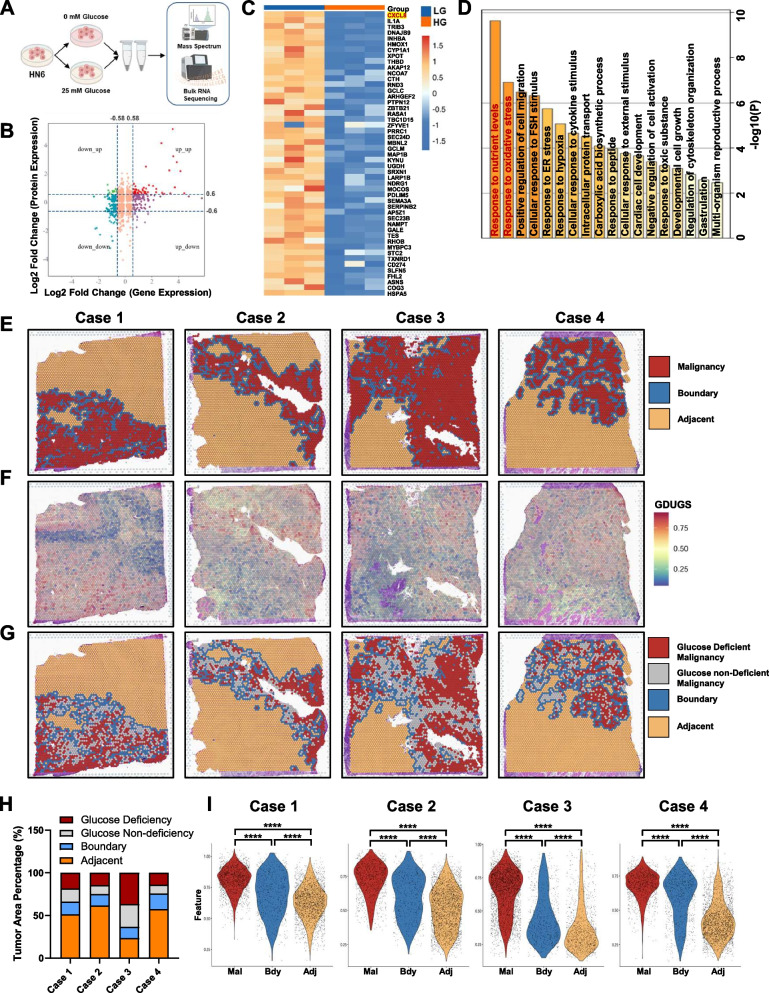
Integrated multiple omics analysis predicts spatial heterogeneity of glucose deficiency in HNSCC. **A** Schematic diagram of integrated analysis on transcriptome and proteomics. **B** Nine-quadrant diagram shows both differentially expressed genes and proteins in HN6 cells treated under indicated conditions for 8 h. **C** Heatmap of upregulated genes set (GDUGS) in both HN6 cells (Foldchange > 1.5, *p* < 0.05). **D** GO enrichment of upregulated gene set. **E** Four tissue sections of HNSCC cases were divided into malignancy, boundary and adjacent based on COTTRAZM analysis. **F** Spatial distribution of GDUGS in four cases. **G**, **H** Four tissue sections of malignant area were divided into glucose deficient and non-deficient malignancy based on GDUGS levels. The proportion of spots in each tissue region is presented, respectively. **I** Violin plot showing that the GDUGS levels in different regions of four HNSCC cases. *p < 0.05, **p < 0.01, ***p < 0.001, ****p < 0.0001

To obtain mechanistic insights into nutrient starvation-induced gene expression in HNSCC, we established an in vitro glucose-deficient HN6 model and collected cell lysates for further RNA-Seq and LC-MS/MS analysis (Fig. 1A). In total, 89 differentially expressed mRNAs and proteins were identified, including 49 upregulated and 40 downregulated genes in glucose-deficient group compared with the normal culture group (Fig. 1B-C and Supplementary Fig. 1A-B). Among the differentially expressed mRNAs and proteins, the glucose deficiency upregulated gene set (GDUGS) in the transcriptome and proteome was particularly interesting for two reasons: (1) the upregulated gene set is easier to detect than the downregulated gene set in HNSCC tissues; (2) the upregulated proteins are more likely to be therapeutic targets for further clinical intervention. Gene Ontology (GO) enrichment of the upregulated genes associated with glucose deficiency revealed that “response to nutrient levels” and “response to oxidative stress” were the most enriched terms (Fig. 1D). These data suggest that the GDUGS reflects the cell response to nutrient-starvation.

The advantages of spatial transcriptomics (ST) combined with single-cell RNA sequencing (scRNA-seq) enable gene expression profiling coupled with two-dimensional spatial information directly within tissues [31]. To identify potential therapeutic targets from highly heterogeneous tumor sections, we selected Cottrazm [30], an integrated tool framework that constructs the microenvironment around the tumor boundary on the basis of spatial transcriptomics data and H&E images (GSE220978 and GSE208253). We observed a clear tumor boundary in four HNSCC tissue samples (Fig. 1E). According to the GDUGS expression levels (Fig. 1F), the malignant tumor area was defined as glucose-deficient and non-deficient area (Fig. 1G-H and Supplementary Fig. 1C). Local spatial gradient inference (LGSI) [31] was adopted to compute spatial gradient of GDUGS expression levels (Fig. 1I). For each patient, GDUGS expression levels were significantly higher in malignant tissues than in adjacent tissues (Fig. 1I). The survival time of patients with high GDUGS levels was significantly shorter than that of patients with low GDUGS expression levels (Supplementary Fig. 1D). These data indicate that the GDUGS is a useful tool for predicting the spatial heterogeneity of glucose deficiency in HNSCC.

### Glucose deficiency promotes CXCL8 expression via ROS-mediated activation of the NFκB pathway in HNSCC

**Fig. 2:**
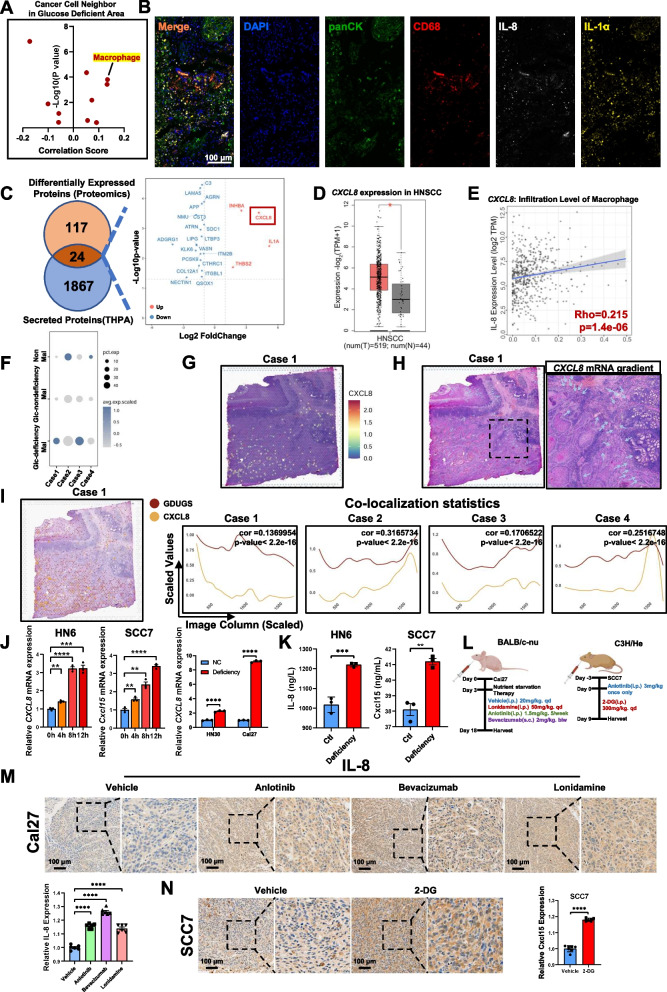
Glucose deficiency induces *CXCL8* expression in HNSCC. **A** Cell neighbor analysis between cancer cells and other cells in the spatial transcriptomics. **B** Representative images of multiplex immunohistochemistry assay on panCK (tumor), CD68 (macrophage), IL-1A and IL-8 (remarkable GDUGS symbol) levels in clinical patients. **C** Volcano plot displays intersection of GDUGS and secreted proteins from THPA database. **D** *CXCL8* mRNA levels in HNSCC and normal tissues from TCGA database. **E** Correlation between *CXCL8* mRNA level and macrophage infiltration in HNSCC from TCGA database. **F** Bubble chart shows *CXCL8* mRNA level in different regions of four cases. **G** Spatial distribution of *CXCL8* mRNA levels in Case 1. **H** Spatial gradient of *CXCL8* mRNA levels in Case 1. **I** Colocalization of *CXCL8* mRNA and GDUGS in four cases. **J** Quantitative PCR analysis on human *CXCL8* and mouse *Cxcl15* mRNAs under glucose-deficiency. **K** ELISA assay on human IL-8 and mouse Cxcl15 proteins under glucose-deficiency. **L** Schematic diagram of establishing Cal27 xenograft into nude mice tongues and SCC7 into C3H/He mice, followed by nutrient-starvation therapies. **M** Immunohistochemistry analysis on human IL-8 levels in Cal27 xenograft tissues. **N** Immunohistochemistry analysis on mouse Cxcl15 levels in SCC7 xenograft tissues. *p < 0.05, **p < 0.01, ***p < 0.001, ****p < 0.0001

To identify the most interactive cell type of cancer cell neighbors, the nearest 6 spots around each Visium spot were considered nearest neighbors, and the DBSCAN [29] algorithm was applied to calculate both molecular and cell neighbors. Tumor-associated macrophages (TAMs) were the cell type with the highest score in the glucose-deficient area of the tumor nest (Fig. 2A and Supplementary Fig. 2A). In the multiplex immunohistochemical staining on 6 tissue samples, TAMs were enriched in the glucose-deficient area of the tumor nest (Fig. 2B and Supplementary File 2). Cancer cells can secrete proteins to instruct or educate TAMs, thereby reshaping the tumor microenvironment [35]. To explore this process, we integrated the GDUGS derived from an in vitro glucose-deficient HN6 model with a secreted protein library (THPA of the Atlas library, Supplementary File 3). We found that the *CXCL8* mRNA and IL-8 protein levels in glucose-deficient HN6 cells were significantly greater than those in non-glucose-deficient cells (Fig. 2C). CXCL8 levels are greater in the tumor tissue of HNSCC patients than in normal tissue, and the survival time of patients with high *CXCL8* mRNA levels was shorter in TCGA data (Fig. 2D and Supplementary Fig. 2B); moreover, CXCL8 was highly correlated with the infiltration of macrophages and clinical pathological stage in TCGA data (Fig. 2E and Supplementary Fig. 2C).

*CXCL8* mRNA-positive puncta were localized mainly in the malignant glucose-deficient areas of HNSCC tissue sections (Fig. 2F). In the analysis on single-cell transcriptomics data (GSE234933), *CXCL8* mRNA was expressed mainly in macrophage and epithelial (Supplementary Fig. 2D). However, macrophages aggregate in the adjacent area and it is the source of CXCL8 outside the tumor boundary (Fig. 2G and Supplementary Fig. 2E, 2H). Moreover, the *CXCL8* mRNA gradient ranged from cancer cells to TAM-enriched areas (Fig. 2H). Colocalization analysis revealed that the GDUGS-defined glucose-deficient area was significantly correlated with CXCL8 expression (Fig. 2I and Supplementary Fig. 2F). These data suggest that *CXCL8* mRNA is highly expressed in the glucose-deficient area of HNSCC tissues.

Subsequently, in vitro qPCR and ELISA experiments were conducted to verify that HNSCC cells significantly upregulated *CXCL8* mRNA and IL-8 under conditions of glucose deficiency. However, a murine ortholog of CXCL8 does not exist [36]. Instead, researchers typically use chemokines such as Cxcl15 as substitutes for IL-8 in mouse models to investigate similar biological effects and functions [37]. We found that mouse-derived HNSCC SCC7 cells and human-derived HNSCC HN6 cells contained higher glucose concentrations and consumed more glucose compared with human normal oral keratinocytes (HOKs), indicating that HN6 and SCC7 cells are glucose deficient at baseline (Supplementary Fig. 2G). In HN6 and SCC7 cells, glucose deficiency significantly upregulated *CXCL8*, *Cxcl15* mRNA and their protein expressions in a dose-dependent manner (Fig. 2J-K). To examine whether glucose deficiency increases IL-8 and Cxcl15 levels in vivo, we evaluated two classes of nutrient starvation therapies: glycolysis inhibitors and angiogenesis inhibitors. To inhibit glycolysis, we used the hexokinase inhibitor Lonidamine [38] and the glucose analog 2-DG [39] in phase II clinical studies. To inhibit angiogenesis, we used the VEGFR2 inhibitor anlotinib [40] and a humanized VEGF antibody, bevacizumab [41]. We subject the CAL27 and SCC7 tongue xenograft models to nutrient starvation (Fig. 2L) and found that each nutrient starvation treatment significantly increased the IL-8 and Cxcl15 levels compared with those in the vehicle group (Fig. 2M, N). These data suggest that glucose deficiency strongly induces *CXCL8* mRNA and IL-8 protein expression in HNSCC tissues.

### Glucose deficiency promotes CXCL8 expression via ROS activating NF-κB pathway in HNSCC

**Fig. 3:**
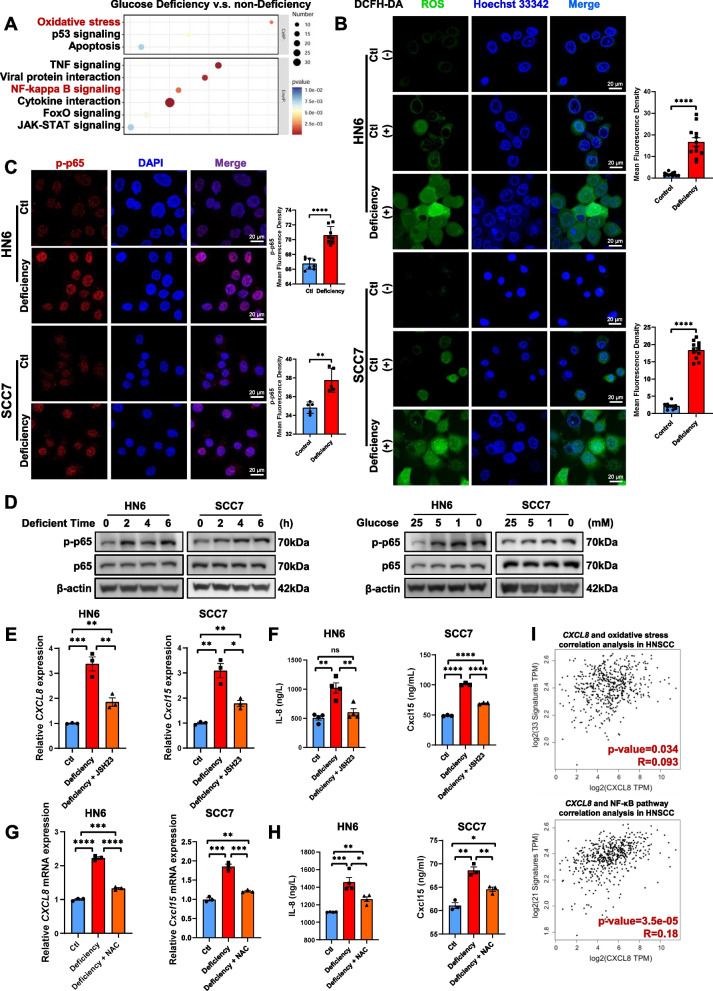
Glucose deficiency promotes *CXCL8* expression via ROS activating NF-κB pathway in HNSCC. **A** KEGG enrichment of differentially expressed genes in transcriptome of HN6 cells under glucose-deficiency. **B** Representative immunofluorescence images and quantification of ROS levels in SCC7 and HN6 cells under glucose-deficiency. **C** Representative immunofluorescence images and quantification of p-NF-κB p65 (Ser 536) location in SCC7 and HN6 cells under glucose-deficiency. **D** Immunoblotting assay on NF-κB p65 and phospho-NF-κB p65 (Ser 536) levels in SCC7 and HN6 cells under different glucose-deficiency conditions. **E** Quantitative PCR analysis on human *CXCL8* and mouse *Cxcl15* mRNAs under glucose-deficiency. Cells were treated with vehicle or 10 μM JSH-23 (NF-κB inhibitor). **F** ELISA assay on human IL-8 and mouse Cxcl15 proteins under glucose-deficiency. Cells were treated with vehicle or 10 μM JSH-23 (NF-κB inhibitor). **G** Quantitative PCR analysis on human *CXCL8* and mouse *Cxcl15* mRNAs under glucose-deficiency. Cells were treated with vehicle or 5 mM NAC (ROS inhibitor). **H** ELISA assay on human IL-8 and mouse Cxcl15 proteins under glucose-deficiency. Cells were treated with vehicle or 5 mM NAC (ROS inhibitor). **I** Correlation between CXCL8 mRNA levels and indicated gene set (Oxidative stress and NF-κB signaling) in HNSCC from TCGA database. *p < 0.05, **p < 0.01, ***p < 0.001, ****p < 0.0001

To determine how glucose deficiency promotes CXCL8 expression, we analyzed the RNA-seq data from an in vitro glucose-deficient HN6 model. Pathway enrichment and further GSEA revealed significantly upregulated expression of genes related to oxidative stress and activation of the NFκB pathway in glucose-deficient HN6 cells compared with control cells (Fig. 3A and Supplementary Fig. 3A, B). In the spatial transcriptome of HNSCC, the malignant area expressed more genes that respond to oxidative stress and activate the NFκB pathway (Supplementary Fig. 3C, D). As glucose deprivation induces cell death by increasing intracellular oxidative stress [42], NF-κB heterodimers may be directly modified under conditions of increased oxidative stress [43]. To examine whether glucose deprivation induces ROS and NF-κB activation, we cultured human HN6 cells and mouse SCC7 cells with or without 25 mM glucose and detected ROS production using a fluorescent probe. Compared with that in the control group under normal conditions, the fluorescence intensity of the ROS probe was significantly greater in the cancer cells in the glucose-deprivation group (Fig. 3B). Phosphorylated p65 (p-NF-κB p65) indicates activation, enabling nuclear translocation and gene transcription, essential for assessing pathway activity [12]. The accumulation of p-p65 significantly increased in the nuclei of both HN6 and SCC7 cells under conditions of glucose deficiency (Fig. 3C). The ratio of p-p65 and p65 protein levels in HN6 cells and SCC7 cells increased according to immunoblotting results (Fig. 3D). These data support that glucose deficiency increases ROS and NF-κB activation.

N-acetylcysteine (NAC), the N-acetyl derivative of the natural amino acid l-cysteine, acts as a reduced antioxidant glutathione precursor to clear ROS [44]. NAC incubation reduced the ratio of p-p65 and p65 protein levels in HN6 cells under glucose deprivation (Supplementary Fig. 3E). To examine whether glucose deficiency increases CXCL8 expression via ROS and NF-κB activation, we examined mRNA and protein levels of CXCL8 and Cxcl15 in HN6 and SCC7 cells with these compounds. Compared with the vehicle, the specific NF-κB inhibitor JSH-23 [45] significantly reduced *CXCL8* and *Cxcl15* mRNA levels in glucose-deficient HN6 cells and SCC7 cells (Fig. 3E). Compared with the vehicle, NAC significantly reduced *CXCL8* and *Cxcl15* levels in glucose-deficient HN6 cells and SCC7 cells (Fig. 3G). Moreover, ELISA revealed that JSH-23 and NAC significantly reduced IL-8 and Cxcl15 protein levels in glucose-deficient HN6 cells and SCC7 cells (Fig. 3F, H). The finding that high *CXCL8* mRNA levels were positively correlated with oxidative stress and NF-κB signaling gene expression was also demonstrated using bioanalysis (Fig. 3I and Supplementary Fig. 3F). GSEA of RNA-seq data from HNSCC patients revealed significant upregulation of the oxidative stress pathway in glucose-deficient areas compared with non-glucose-deficient areas (Supplementary Fig. 3G). These data suggest that glucose deficiency promotes CXCL8 and Cxcl15 expression via ROS-mediated activation of the NF-κB pathway in HNSCC.

### Cancer cell-derived IL-8 induces CLU expression in macrophages to facilitate antioxidation in cancer cells

**Fig. 4:**
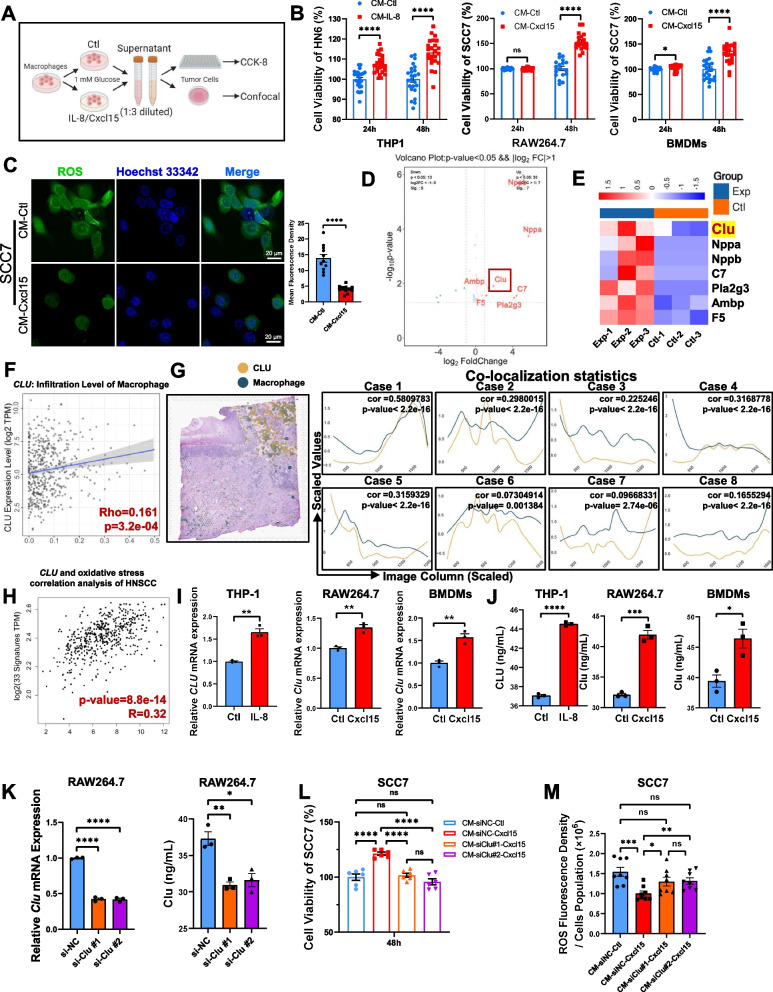
IL-8 induces CLU expression in TAMs to facilitate anti-oxidation in cancer cells reversely. **A** Schematic diagram of macrophages supernatant production process followed by tumor cells treatment and analysis. **B** CCK-8 assay on cell viability after cultured with macrophage conditional media. **C** Representative immunofluorescence images and quantification of ROS levels in SCC7 cells after treated with indicated macrophage conditional media. **D**, **E** The volcano plot and heatmap of differentially expressed genes in BMDMs stimulated by recombinant mouse Cxcl15 protein intersected with genes encoding secreted proteins from THPA database. **F** Correlation between *CLU* mRNA levels and macrophages infiltration in HNSCC from TCGA database. **G** Colocalization of the spatial distribution of *CLU* and macrophages in four main cases and four supplementary cases. **H** Correlation between *CLU* mRNA levels and Oxidative stress gene-set in HNSCC from TCGA database. **I** Quantitative PCR analysis on human *CLU* and mouse *Clu* mRNA in macrophages treated with recombinant human IL-8 or mouse Cxcl15. **J** ELISA assay on human CLU and mouse Clu proteins in macrophages treated with recombinant human IL-8 or recombinant Cxcl15. **K** Quantitative PCR analysis on *Clu* mRNA and ELISA assay on secreted Clu protein of RAW264.7 cell treated by si-NC or si-Clu#1, si-Clu#2. **L** CCK-8 assay on cell viability after cultured with indicated macrophage conditional media. **M** Immunofluorescense on ROS levels in SCC7 cells after cultured with indicated macrophage conditional media. *p < 0.05, **p < 0.01, ***p < 0.001, ****p < 0.0001

The CCK-8 (Cell Counting Kit-8) assay is a colorimetric method used to measure cell viability. To verify whether IL-8 modulates macrophage function to increase cancer cell viability under conditions of glucose deficiency, we cultured cancer cells in conditioned medium obtained from macrophages treated with IL-8, Cxcl15 or the control and conducted CCK-8 experiments to detect the activity of cancer cells (Fig. 4A). We used BMDMs (primary murine macrophages), RAW264.7 (murine macrophage cell line), and THP-1 (human monocytic cell line) to explore macrophage and monocyte responses. BMDMs provide physiological relevance (Supplementary Fig. 4A), RAW264.7 ensures reproducibility, and THP-1 bridges findings to human biology, offering a comprehensive understanding of chemokine effects across species. Compared with those from the control group, the supernatants obtained from macrophages stimulated with the IL-8 or Cxcl15 protein significantly increased cancer cell viability under conditions of glucose deficiency (Fig. 4B). Moreover, the supernatant of macrophages stimulated with the Cxcl15 protein significantly decreased ROS production in cancer cells (Fig. 4C). These data suggest that the use of IL-8 or its substitute Cxcl15 in mouse models improves the tolerance of cancer cells to glucose deficiency via the modulation of macrophage function.

To identify which macrophage-derived cytokines improve cancer cell tolerance to glucose deficiency, we conducted high-throughput sequencing of RNA-seq data from Cxcl15-treated BMDMs (Fig. 4D). Through integrated analysis of significantly upregulated genes with secretion protein libraries in public databases, we obtained 7 candidate cytokines (Fig. 4D). Clu was the only factor reported to be a pivotal cytokine that negatively regulated tumor cell apoptosis under severe conditions (Fig. 4E). Clu is a protein involved in various cellular processes, including apoptosis, cell survival, and stress response [16]. Analysis of the spatial transcriptome indicated that *CLU* mRNA levels were significantly correlated with macrophage distribution in HNSCC samples from the TCGA database (Fig. 4F), and this finding was also supported by bioanalysis and colocalization analysis (Fig. 4G and Supplementary Fig. 4B, C). Moreover, there was a correlation analysis between CLU expression and oxidative stress signatures in HNSCC samples from the TCGA database. Pearson correlation was used, with a correlation coefficient (R) of 0.32 and a highly significant *p*-value of 8.8e-14 (Fig. 4H). These data suggest that IL-8 promotes CLU expression in macrophages.

Subsequently, to determine whether IL-8 or Cxcl15 protein can stimulate CLU expression in macrophages, To determine whether the IL-8 or Cxcl15 protein stimulates CLU expression in macrophages, human THP-1 cells were subsequently incubated with IL-8, whereas mouse RAW264.7 cells and BMDMs were treated with Cxcl15 for 24 h. Compared with vehicle treatment, IL-8 or Cxcl15 treatment significantly induced the expression of human *CLU* mRNA or mouse *Clu* mRNA, as determined via a qPCR assay (Fig. 4I). IL-8 and Cxcl15 treatment also significantly increased the level of the CLU and Clu protein in THP-1, RAW264.7 cells and BMDMs, as shown by ELISA (Fig. 4J), suggesting that IL-8 increases *CLU* mRNA and protein expression. Supplement of recombinant human CLU significantly increased cell viability of HN6 and SCC7 cells under glucose-deficiency (Supplementary Fig. 4D). To examine whether macrophage-derived Clu promotes cancer cell viability, mouse RAW264.7 cells were treated with si-NC, si-Clu-1 or si-Clu-2. Both siRNAs against Clu significantly decreased *Clu* mRNA and protein levels in RAW264.7 cells (Fig. 4K). Next, we cultured cancer cells in the conditioned medium obtained from macrophages treated with Cxcl15 or the control and conducted CCK-8 experiments to detect tumor cell activity (Fig. 4L). Compared with those from the si-NC group, the supernatants from the si-Clu-treated macrophages failed to increase cancer cell viability under conditions of glucose deficiency (Fig. 4L). ROS detection also revealed that the supernatant from si-Clu-treated macrophages did not reduce ROS production, whereas that from the si-NC group did (Fig. 4M). These data indicate that IL-8 or its substitute Cxcl15 promotes CLU expression in macrophages to facilitate antioxidative stress in cancer cells under conditions of glucose deficiency.

### Pharmacological blockade of CXCL8 signaling enhances the antitumor effect of nutrient-starvation therapies in vivo

**Fig. 5:**
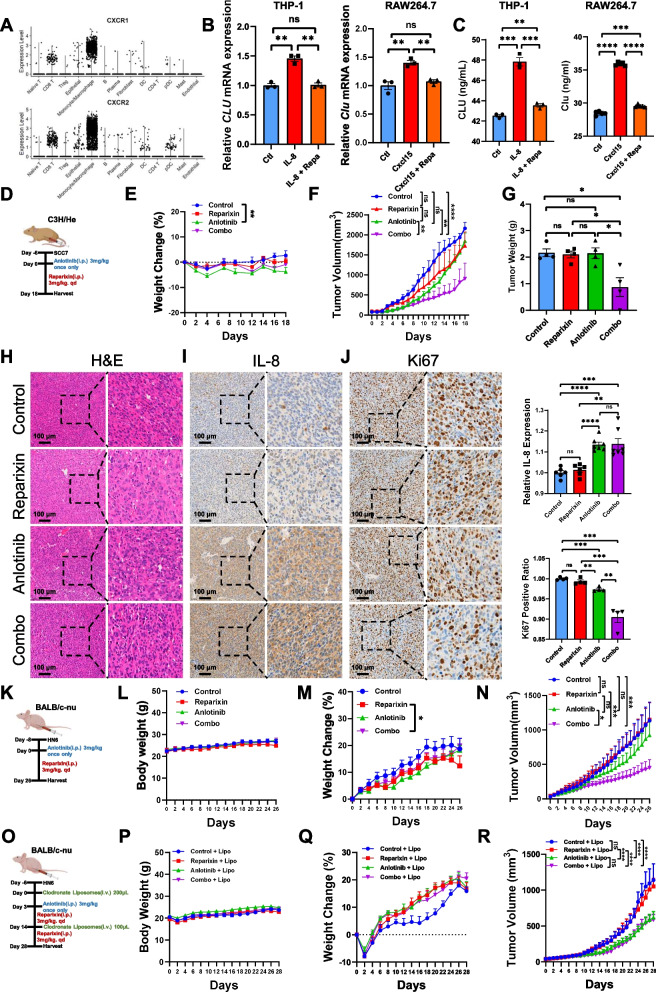
Pharmalogical blockade of *CXCL8* signaling enhances the antitumor effect of nutrient starvation therapies in vivo. **A** *CXCR1* and *CXCR2* mRNA of tumor-infiltrating cells from scRNA-seq data. **B** Quantitative PCR analysis on human *CLU* and mouse *Clu* mRNA in macrophages stimulated with recombinant human IL-8 or mouse Cxcl15. Cells were treated with vehicle or 10 μM reparixin (CXCR1/2 inhibitor). **C** ELISA assay on human CLU and mouse Clu proteins in macrophages stimulated with recombinant human IL-8 or recombinant Cxcl15. Cells were treated with vehicle or 10 μM reparixin (CXCR1/2 inhibitor). **D** Schematic diagram of SCC7 cells flank implantation and medicine treatment (*n* = 5). **E** Quantification of weight change after mice were treated with vehicle, anlotinib, reparixin or combination. **F** Quantification of tumor volumn at indicated days. **G** Quantification of tumor weight at the end of treatment. **H** Representative images of H&E staining in SCC7 xenografts after indicated treatment. **I** Representative images and quantification of immunohistochemistry assay on IL-8 level. **J** Representative images and quantification of immunohistochemistry assay on Ki67 level. **K** Schematic diagram of HN6 cells flank implantation and medicine administration (*n* = 6). **L** Quantification of body weight at indicated days. **M** Quantification of weight change at indicated days. **N** Quantification of tumor volume at indicated days. **O** Schematic diagram of HN6 cells flank implantation and medicine administration with macrophage depletion (*n* = 8). **P** Quantification of body weight at indicated days. **Q** Quantification of weight change at indicated days. **R** Quantification of tumor volume at indicated days.*p < 0.05, **p < 0.01, ***p < 0.001, ****p < 0.0001

Reparexin is a noncompetitive allosteric inhibitor of the IL-8 receptors CXCR1 and CXCR2, and its cytotoxic effects on cancer cells at different concentrations were not significantly different (Supplementary Fig. 5A). We analyzed publicly available single-cell data from HNSCC patients (GSE234933) to validate which cell types are specifically associated with the IL-8 receptors CXCR1 and CXCR2 in the tumor microenvironment of HNSCC patients. We found that CXCR1 and CXCR2 are expressed mainly in T cells and monocytes/macrophages (Fig. 5A and Supplementary Fig. 5B-D). Indeed, reparixin significantly inhibited IL-8 induced *CLU* mRNA and CLU protein in THP-1 cells, as well as suppression on Cxcl15 induced *Clu* mRNA and Clu protein in RAW264.7 cells (Fig. 5B, C). These data suggesting that reparixin can also inhibit IL-8 signaling in macrophages without influence on cell viability of cancer cells.

Anlotinib is a clinically used nutrient-starvation agent that inhibits tumor angiogenesis and nutrient supply by targeting VEGFR, FGFR, and PDGFR through tyrosine kinase activity inhibition [46]. Inspired by in vitro data showing that si-CLU reduced the antioxidative stress capability and inhibited HNSCC cell viability under conditions of glucose deficiency, we further analyzed whether inhibiting CXCL8 signaling enhances the efficacy of anlotinib in suppressing HNSCC tumor growth. We established an SCC7 xenograft model in which C3H mice were separated into four treatment groups: vehicle, anlotinib alone, reparixin alone, and a combination of anlotinib and reparixin (Fig. 5D). No significant difference in body weight was noted among the four groups (Fig. 5E). Compared with those in the vehicle group, the tumors in the reparixin and anlotinib groups were smaller, and the tumors in the combination group were the smallest among the four groups (Fig. 5F and G). Immunohistochemical analysis of IL-8 levels revealed that the intensity of the anlotinib-treated and combination-treated tumors was significantly greater than that of the vehicle-treated tumors, whereas the intensity of the reparixin-treated tumors was not significantly different from that of the vehicle-treated tumors (Fig. 5H, I). Compared with that in the vehicle group, Ki67 expression in the reparixin-treated tumors was not significantly different, whereas that in the anlotinib-treated tumors was significantly lower. Ki67 expression was the lowest in the combination-treated tumors among the four groups (Fig. 5J). Compared with that in the vehicle group and the reparixin group, Clu expression significantly increased in the anlotinib-treated tumors, whereas that in the combination-treated tumors significantly decreased (Supplementary Fig. 5E).These data indicate the synergistic antitumor effect of reparixin when it blocks CXCL8 signaling in combination with nutrient-starvation therapy.

To examine whether anlotinib and reparixin synergistically inhibited tumor growth in T-cell-deficient nude mice, we established another HN6 xenograft model and administered four different therapies in nude mice (Fig. 5K). Notably, nude mice still have no normal T and B cells, but normal macrophages. No significant difference in body weight was noted between the vehicle and combination groups (Fig. 5L, M). Compared with those in the vehicle group, the tumors in the reparixin and anlotinib groups were smaller, and the tumors in the combination group were the smallest among the four groups (Fig. 5N). These data suggest that pharmacological blockade of CXCL8 signaling enhances the antitumor effect of nutrient-starvation therapies without T cell involved. To further examine whether CXCL8 signaling blockade enhances the antitumor effect of nutrient starvation therapies via modulating TAMs, mice were injected i.p. with 200 μl of liposomal clodronate on days 7 after tumor inoculation and then 100 μl every two weeks for macrophage depletion (Fig. 5O). No significant difference in body weight was noted also (Fig. 5P, Q). While Anlotinib + Lipo and Combo + Lipo are more effective in controlling tumor growth compared to Saline + Lipo and Reparixin + Lipo, there is no significant difference in efficacy between Anlotinib + Lipo and the combination treatment (Fig. 5R). These data support that CXCL8 signaling blockade enhances the antitumor effects of nutrient-starvation therapies via TAM modulation.

### A gene set that is upregulated under conditions of glucose deficiency is positively correlated with poor outcomes in multiple types of cancer patients

**Fig. 6:**
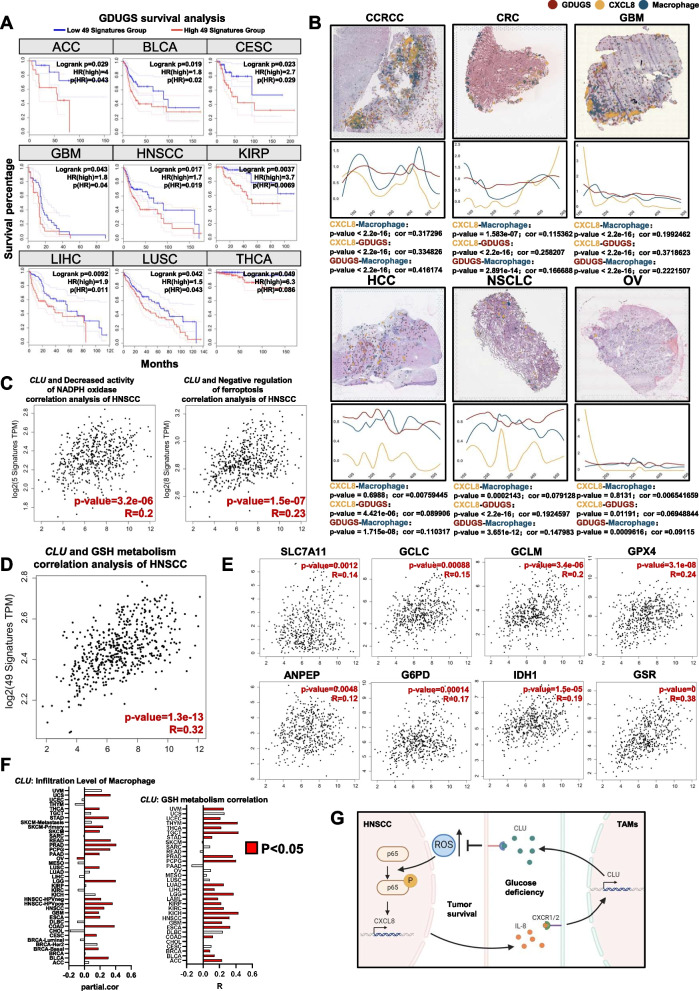
Upregulated gene set under glucose deficiency positively correlates with poor outcomes in multiple types of cancer patients. **A** Correlation between the expression levels of GDUGS and survival duration in various cancer from TCGA database. **B** Colocalization of the spatial distribution of GDUGS, *CXCL8* mRNA and macrophages in various cancers, and the correlation in various cancers is presented, respectively. **C** Correlation between the expression levels of *CLU* and decreased activity of NADPH oxidase and negative regulation of ferroptosis gene-set in HNSCC from TCGA database. **D** Correlation between the expression levels of *CLU* and GSH metabolism in HNSCC from TCGA database. **E** Correlation between *CLU* expression levels and human GSH metabolism genes (including SLC7A11, GCLC, GCLM, GPX4, ANPEP, G6PD, IDH1, GSR) in HNSCC from TCGA database. **F** The correlation between *CLU* and macrophages infiltration, GSH metabolism in various cancer. **G** Schematic representation of tumor-derived CXCL8 educates tumor associated macrophages to counteract oxidative stress under glucose-deficiency

To determine the expression levels of the genes in the GDUGS and their potential correlation with poor prognosis, data from patients with various cancer types in TCGA database were analyzed. High GDUGS mRNA levels were associated with poorer prognosis (Fig. 6A). Moreover, colocalization statistics revealed that GDUGS-defined glucose deficiency, CXCL8 expression and macrophage distribution were significantly correlated (Fig. 6B), suggesting that cancer cell-derived IL-8 may induce CLU expression in TAMs to facilitate antioxidation in various types of cancer. Notably, *CLU* mRNA levels were highly correlated with reduced NADPH oxidase activity and negative regulation of ferroptosis in HNSCC (Fig. 6C), indicating that CLU potentially reduces ROS production in cancer cells. To confirm this hypothesis, we conducted a bioanalysis of data from TCGA database and discovered that *CLU* mRNA levels were significantly correlated with glutathione metabolism gene expression in HNSCC (Fig. 6D and E), and the qPCR assay data in vitro were in accord with bioanalysis results (Supplementary Fig. 6A, B) Furthermore, our data revealed that *CLU* mRNA levels play critical roles in macrophage infiltration and GSH metabolism in various cancers (Fig. 6F).

Overall, our results demonstrate the existence of a feedback loop between cancer cells and glucose-deficient regions in TAMs. HNSCC-derived CXCL8 favors endogenous antioxidative processes and confers therapeutic resistance to nutrient starvation in HNSCC. Importantly, this feedback loop may occur in other types of cancer (Fig. 6G).

## Discussion

In this study, we identified an upregulated gene set involved in glucose deficiency using high-throughput screening. Mapping these genes to spatial transcriptomic data further provides deeper insight into the regulatory mechanisms of antioxidative stress caused by glucose deficiency as well as new insights into disease diagnosis and treatment. This approach not only aids in understanding the regulatory mechanisms of glucose metabolism but also provides new research tools and directions for biomedical studies, driving progress in related fields. Both high-throughput screening and spatial transcriptomic data generate large and complex datasets; therefore, interpreting these data requires specialized bioinformatics knowledge and skills.

HPV is more closely related to oropharynx tissue or other head and neck cancer subsites. HPV accounts for only 2–5% of OSCC cases, and the significance of HPV infection in OSCC is currently unknown. In some cases, the determination of HPV infection is not mandatory. OSCC tissue samples were obtained from 4 patients in GSE220978 [47]. In these cases, the direct pathogenic factor was oral submucous fibrosis caused by betel nut chewing, and HPV infection status was not assessed. We included additional OSCC spatial transcriptomics data from the GSE208253 dataset [48], and the patients were all HPV negative. It has been reported that patients with HPV + OSCC or oropharyngeal squamous cell carcinoma (OPSCC) typically have a better prognosis [49], and we will fully consider the effect of HPV infection in future studies.

We chose glucose deficiency upregulated gene set (GDUGS) in the transcriptome and proteome because upregulated genes often exhibit higher expression levels, making them more readily detectable by commonly used molecular assays such as RNA sequencing or microarray analysis. Additionally, upregulated genes may have more pronounced effects on cellular functions and pathways, leading to more noticeable phenotypic changes that can be observed histologically or through other experimental approaches. Therefore, we suggest that the detection of upregulated gene sets may be more straightforward and clinically relevant in the context of HNSCC. Regarding genes that are regulated differently at the RNA and protein levels on their findings, post-transcriptional and post-translational regulatory mechanisms can exert significant and crucial influences on the processes of gene expression and protein function. Future research endeavors may indeed be justified and necessary to delve into the potential implications and consequences of genes that undergo differential regulation at the RNA and protein levels.

We observed a notable concentration of GDUGS in malignant areas. It is crucial to delve into whether this phenomenon is attributed to the signature being generated specifically within cancer cells and its subsequent inability to predict the starvation status of adjacent tissues. Cancer cells are known for their altered gene expression profiles, which are often driven by genetic mutations, epigenetic changes, and deregulation of signaling pathways. It is plausible that the GDUGS reflects these cancer-specific alterations, serving as a biomarker or a set of genes that are differentially expressed in response to the tumor microenvironment. This discrepancy could arise from differences in gene expression patterns, metabolic pathways, or signaling networks between cancer cells and their adjacent counterparts. Therefore, exclusively relying on the GDUGS to infer the starvation status of adjacent tissues may lead to misinterpretation of the data. Further research is needed to explore the intricate relationships among the GDUGS, cancer cells, and their microenvironments. This could involve comprehensive gene expression profiling of different cell types within the tumor microenvironment, as well as investigating the metabolic pathways and signaling networks that are perturbed in response to tumor growth and starvation.

The CXCL8/IL-8 axis, which has been studied most extensively for its role in proinflammatory processes, has been implicated in tumor growth and metastasis in several cancers. However, the role of IL-8 in the tolerance of tumors to glucose deficiency has not been elucidated in previous studies. Therefore, the focus of this study was to explore the specific molecular mechanism by which IL-8, which is highly expressed in HNSCC under conditions of glucose deficiency, enhances tumor cell resistance to glucose deficiency and to provide new therapeutic ideas to address drug resistance in nutrient-starvation therapy. One of the main effective cell types associated with the CXCL8/IL-8 axis is macrophages, especially tumor-associated macrophages (TAMs), which play crucial roles in disrupting the progression of cancer through various mechanisms, including cytokine secretion and immunosuppression. Macrophages aggregate in glucose-deficient areas. However, outside of the tumor boundary, macrophages aggregate near the infiltrating margin due to the barrier effects of the tumor. These factors contribute to the discrepancy between the proximity of macrophages to glucose-deficient tumor areas.

Glucose is the primary energy source for cells. Alterations in glucose levels can cause an imbalance in the production and clearance of reactive oxygen species (ROS), leading to an accumulation of ROS and subsequent oxidative stress in various cancers, particularly HNSCC, because of its high aerobic glycolytic activity. The increased production of ROS in the absence of sufficient glucose can damage cellular components, including DNA, proteins, and lipids, further contributing to oxidative stress and leading to cell apoptosis. Tumor cells tolerate this strict environment by enhancing protective cell autophagy, altering primary energy sources such as lactate and glutamine, or inducing surrounding immune cells to secrete antioxidants to protect against excessive oxidative stress damage. Clusterin (CLU) is a sulfated chaperonic glycoprotein that is expressed in all tissues and human fluids and is involved in diverse cellular functions, including cell cycle regulation, apoptotic cell death, DNA repair, membrane recycling, lipid transportation, and immune system regulation [16]. Notably, CLU exhibits promising potential as a biomarker for HNSCC [18, 50]. The CXCL8/IL-8/CLU axis was first described in this study. Importantly, we demonstrated that HNSCC-derived CXCL8 induced by glucose deficiency can stimulate TAMs to synthesize and secrete CLU to counteract oxidative stress in HNSCC.

The application of reparixin in antitumor research is primarily related to its role as a potent and specific allosteric inhibitor of the CXCL8 receptors CXCR1 and CXCR2. Specifically, reparixin inhibits CXCL8-induced biological activities, strongly blocking CXCR1/CXCR2-mediated inflammation. In antitumor research, reparixin may be used to prevent cancer cell migration and proliferation because it interferes with signal communication between tumor cells and their surrounding environment, potentially slowing tumor growth and spread. Although reparixin has been shown to have inhibitory effects on tumor cells under laboratory conditions, further research and clinical trials are needed to verify its application and efficacy in actual clinical treatment.

The importance of the synergistic effect of reparixin and anlotinib in significantly inhibiting the growth of HNSCC in two mouse xenograft models lies in several aspects. This synergistic action suggests that these two drugs can target tumors through multiple mechanisms, thereby enhancing the therapeutic outcome. This synergistic effect also provides scientists with a window to explore new strategies for cancer treatment, potentially leading to the development of novel drug combinations or therapeutic approaches. Although the synergistic effect may reduce the side effects of individual drugs, it may also introduce new, unknown side effects that require close monitoring. Different drugs may interact with each other in terms of metabolism and excretion, and further research is needed to ensure their safety and effectiveness.

However, CXCR1 and CXCR2 are also activated by other ligands, including CXCL1, CXCL2, CXCL3, and CXCL5, among others, which could potentially contribute to the observed therapeutic outcomes. Although our study primarily focuses on CXCL8, the involvement of other chemokines is indeed relevant to fully understanding the signaling and biological effects mediated by CXCR1/2. Future investigations should explore the relative contributions of these chemokines using specific inhibitors or knockdown approaches.

## Conclusions

Our findings provide evidence that the levels of the cancer cell-derived CXCL8 and its encoded protein IL-8 are increased under conditions of glucose deficiency to educate TAMs, facilitate antioxidative mechanisms, induce resistance to nutrient-starvation therapies, and serve as a therapeutic target for overcoming anlotinib resistance in HNSCC patients.

## Supplementary Information

Supplementary Material 1.

Supplementary Material 2.

Supplementary Material 3.

Supplementary Material 4.

## Data Availability

All data supporting the findings of this study are available within the paper and its Supplementary Information.

## Abbreviations

2-DG: 2-Deoxy-D-glucose
ACC: Adrenocortical carcinoma
ANPEP: Alanyl aminopeptidase, membrane
BLCA: Bladder Urothelial Carcinoma
CCRCC: Clear cell renal carcinoma
CESC: Cervical squamous cell carcinoma and endocervical adenocarcinoma
CLU: Clusterin
CM: Conditioned medium
CRC: Colorectal cancer
CXCL8: C-X-C motif chemokine ligand 8
CXCR1/2: C-X-C motif chemokine receptor 1/2
ELISA: Enzyme linked immunosorbent assay
G6PD: Glucose-6-phosphate dehydrogenase
GBM: Glioblastoma
GCLC: Glutamate-cysteine ligase catalytic subunit
GCLM: Glutamate-cysteine ligase modifier subunit
GPX4: Glutathione peroxidase 4
GSH: Glutathione
GSR: Glutathione-disulfide reductase
H&E: Hematoxylin-eosin staining
HCC: Hepatocellular carcinoma
HNSCC: Head and neck squamous cell carcinoma
IDH1: Isocitrate dehydrogenase (NADP( +)) 1
KIRP: Kidney renal papillary cell carcinoma
LIHC: Liver hepatocellular carcinoma
LUSC: Lung squamous cell carcinoma
NAC: N-Acetyl-L-cysteine (N-Acetylcysteine)
NF-κB: Nuclear factor kappa B
NSCLC: Non-small cell lung cancer
OS: Overall survival
OV: Ovarian serous cystadenocarcinoma
PMA: Phorbol 12-myristate 13-acetate
qRT-PCR: Quantitative real-time polymerase chain reaction
ROS: Reactive oxygen species
siRNA: Small interfering RNA
SLC7A11: Solute carrier family 7 member 11
ST: Spatial transcriptomics
TAMs: Tumor-associated macrophages
THCA: Thyroid carcinoma
