# Supplementary material for: Targeting CXCL8 signaling sensitizes HNSCC to anlotinib by reducing tumor-associated macrophage-derived CLU
Source: J Exp Clin Cancer Res. 2025 Feb 5;44:39. doi: 10.1186/s13046-025-03298-7 (PMC11796229; doi:10.1186/s13046-025-03298-7)
Supplement: Supplementary file 1 — Supplementary Material 1. [file 13046_2025_3298_MOESM1_ESM.docx]

**Supplementary Files**

**Supplementary Figure S1.**

**(A)** Heatmap dispalys significantly down-regulated genes set in both HN6 cells transcriptomics and proteomics (Foldchange<0.66, p<0.05).

**(B)** GO enrichment analysis of significantly down-regulated genes both in HN6 cells transcriptomics and proteomics (Foldchange<0.66, p<0.05).

**(C) Four supplementary tissue sections of malignant area were divided into glucose deficient and non-deficient malignancy based on GDUGS levels.**

**(D)** Correlation between the expression levels of UPGS and survival duration in HNSC from TCGA database.

**Supplementary Figure S2.**

**(A)** Cell single plot analysis of the correlation between cancer cells and other cells in the spatial transcriptomics.

**(B)** Correlation of *CXCL8* mRNA and survival duration in HNSC from TCGA database.

**(C)** Correlation of *CXCL8* RNA level and clinical pathological stages in HNSC from TCGA database.

**(D) CXCL8 mRNA of tumor-infiltrating cells from scRNA-seq data.**

**(E)** Spatial distribution of *CXCL8* in the other three cases.

**(F)** Colocalization of the spatial distribution of *CXCL8* and GDUGS in the other three cases.

**(G) The glucose content of cells and supernatant cultured 24 h were detected.**

**Supplementary Figure S3.**

**(A)** GSEA analysis of differentially expressed genes in HN6 cells cultured under glucose-deficiency.

**(B)** Correlation of *CXCL8* RNA level and NF-κB signaling pathway in HNSC from TCGA database.

**(C)** Spatial distribution of oxidative stress genes in the four cases.

**(D)** Spatial distribution of NF-κB signaling genes in the four cases.

**(E) Immunoblotting assay on NF-κB p65 and phospho-NF-κB p65 levels in HN6 cells treated with 5 mM acetylcysteine (NAC) under glucose-deficiency.**

**(F) Correlation between *CXCL8* and *RELA* mRNA levels in HNSCC from TCGA database.**

**(G) GSEA analysis of GDUGS-predicted glucose-deficient malignancy versus non-deficient malignancy from spatial transcriptomics.**

***p<0.05, **p<0.01, ***p<0.001, ****p<0.0001.**

**Supplementary Figure S4.**

**(A)** Gating strategy of primary murine BMDMs (Live+ CD11b+ F4/80+) in wild type mice.

**(B)** Spatial distribution of *CLU* mRNA in four cases.

**(C)** Colocalization of the spatial distribution of *CLU* and macrophages in the other three cases and four supplementary cases.

**(D)** CCK-8 assay on HN6 and SCC7 cells viability after treated with recombinant human CLU under glucose-deficiency.

**Supplementary Figure S5.** (A) CCK-8 assay on cell viability after treated with Reparixin.

(B) UMAP visualization of single-cell transcriptomic profiles showing 13 cell clusters of tumor-infiltrating cells sorted from clinical HNSCC patient’s samples.

(C) UMAP plots and the proportions of tumor-infiltrating cells mainly expressing CXCR1 and CXCR2 mRNA from scRNA-seq data.

(D) Bubble chart displays the expression level of *CXCR1* and *CXCR2* of all kinds of tumor-infiltrating cells from scRNA-seq data.

(E) Representative images and quantification of multiplex immunohistochemistry assay on Clu and macrophage levels in SCC7 xenografts after indicated treatment (n = 10 fields from 5 mice per group).

***p<0.05, **p<0.01, ***p<0.001, ****p<0.0001.**

**Supplementary Figure S6.**

(A) Quantitative PCR analysis on human GSH metabolism genes (including SLC7A11, GCLC, GCLM, GPX4, ANPEP, G6PD, IDH1, GSR) in HN6 and HN30 cells treated with recombinant human CLU under glucose-deficiency.

**Supplementary Table1**

| **Primer name** | **Forward sequence** | **Reverse sequence** |
| --- | --- | --- |
| ACTB | GCCTCGCCTTTGCCGATCC | GCGCGGCGATATCATCATCCA |
| CLU | GAGCGCAAGACACTGCTCA | TTCCCTGGTCTCATTTAGGGC |
| CXCL8 | ACTGAGAGTGATTGAGAGTGGAC | AACCCTCTGCACCCAGTTTTC |
| SLC7A11 | TCTCCAAAGGAGGTTACCTGC | AGACTCCCCTCAGTAAAGTGAC |
| GCLC | GGAGGAAACCAAGCGCCAT | CTTGACGGCGTGGTAGATGT |
| GCLM | TGTCTTGGAATGCACTGTATCTC | CCCAGTAAGGCTGTAAATGCTC |
| GPX4 | GAGGCAAGACCGAAGTAAACTAC | CCGAACTGGTTACACGGGAA |
| ANPEP | TTCAACATCACGCTTATCCACC | AGTCGAACTCACTGACAATGAAG |
| G6PD | ACCGCATCGACCACTACCT | TGGGGCCGAAGATCCTGTT |
| IDH1 | AGAAGCATAATGTTGGCGTCA | CGTATGGTGCCATTTGGTGATT |
| GSR | CACTTGCGTGAATGTTGGATG | TGGGATCACTCGTGAAGGCT |
| Actb | GGCTGTATTCCCCTCCATCG | CCAGTTGGTAACAATGCCATGT |
| Cxcl15 | TGTTGAGCATGAAAAGCCTCTAT | AGGTCTCCCGAATTGGAAAGG |
| Clu | AGCAGGAGGTCTCTGACAATG | GGCTTCCTCTAAACTGTTGAGC |
| **SiRNA name** | **Oligonucleotides** |  |
| *Clu* siRNA-1 | GGGAGUAGGUAUAUUAAUATT tt | UAUUAAUAUACCUACUCCCTT tt |
| *Clu* siRNA-2 | CAGCCUUUCUUUGAGAUGATT tt | UCAUCUCAAAGAAAGGCUGTT tt |

**Supplementary Table2**

| **Antibodies** | **Source** | **Cat** | **Applications** |
| --- | --- | --- | --- |
| Anti-IL-8 | Proteintech | 27095-1-AP | IHC (1:100) |
| Anti-Clusterin  Anti-IL-1A | Abclonal  Abclonal | A13479  A1316 | IF (1:50)  IF (1:50) |
| Anti-β-actin | Abclonal | AC026 | immunoblot (1:50000) |
| Anti-NF-kB p65/RelA | Abclonal | A19653 | immunoblot (1:5000) |
| Anti-pSer536-NF-kB p65/RelA | Abclonal | AP0124 | immunoblot (1:2000), ICC (1:50) |
| Anti-Ki67  Anti-CD68 | Proteintech  Proteintech | 27309-1-AP  28058-1-AP | IHC (1:2000)  IF（1:500） |
| Anti-pan-CK | Proteintech | 26411-1-AP | IF（1:500） |
| Goat Anti-Rabbit IgG(H+L) (Alexa Fluor 647) | Beyotime | Beyotime | ICC (1:500) |
| HRP-labeled Goat Anti-Rabbit IgG (H+L) | Beyotime | A0208 | immunoblot (1:1000), IHC (1:50) |

| **Cell lines** | **Source** | **Cat** |
| --- | --- | --- |
| HOK  HN6 | ScienCellUniversity of Maryland Dental School | Cat# ScienCell-2610; RRID: CVCL_YE19  N/A; RRID: CVCL_5516 |
| HN30 | University of Maryland Dental School | N/A; RRID: CVCL_5525 |
| CAL27 | ATCC | Cat# ATCC-CRL-2095; RRID: CVCL_1107 |
| SCC7 | Bluef (Shanghai) Biotechnology | Cat# BFN60807565; RRID: CVCL_V412 |
| RAW264.7 | ATCC | Cat# ATCC-TIB-71; RRID: CVCL_0493 |
| THP-1 | ATCC | Cat# ATCC-TIB-202; RRID: CVCL_0006 |

| **Experimental animals** | **Source** | **Cat** |
| --- | --- | --- |
| BALB/c nude mice | Shanghai Laboratory Animal Center | N/A |
| C3H/HeJ | Shanghai Laboratory Animal Center | N/A |

| **Chemicals and recombinant proteins** | **Source** | **Cat** |
| --- | --- | --- |
| Reparixin | FEIYUBIO | 266359-83-5 |
| Anlotinib | Selleck | S8726 |
| JSH-23  Acetylcysteine (NAC) | Abmole  MCE | M2786  HY-B0215 |
| Clodronate Liposomes  Recombinant Human IL-8/CXCL8 Protein | SunLipo NanoTech  Sino Biological | SN-ML-E020  10098-HNCH1-10 |
| Recombinant Mouse CXCL15/Lungkine  Recombinant Human Clusterin | NovoProtein  NovoProtein | CJ85  C454 |

| **Critical commercial kits** | **Source** | **Cat** |
| --- | --- | --- |
| Human IL-8 ELISA kit | Hengyuan Biological Technology | HB1944-Hu |
| Human CLU ELISA kit | Hengyuan Biological Technology | HB887-Hu |
| Mouse Cxcl15 ELISA kit | Hengyuan Biological Technology |  |
| Mouse Clu ELISA kit | Hengyuan Biological Technology | HB509-Mu |
| Lipofectamine RNAiMAX | Thermo Fisher | 13778150 |
| BCA Protein Assay Kit | Thermo Fisher | 23227 |
| Reactive Oxygen Species Assay Kit | Beyotime | S0033S |
| Cell Counting Kit-8 | NCM Biotech | C6005 |
| TRIzol Reagent | Takara | 9108 |
| PrimeScript™ RT reagent Kit (Perfect Real Time) | Takara | RR037A |
| Hieff UNICON® qPCR SYBR Green Master Mix(High Rox)  Glucose Assay Kit with GOD-POD  Reactive Oxygen Species Assay Kit | Yeasen  Yeasen  Beyotime | 11200ES08  60408ES60  S0033S |

| **Reagents and consumables** | **Source** | **Cat** |
| --- | --- | --- |
| Fetal Bovine Serum | CellMax | SA211.02 |
| DMEM containing 25mM glucose | BasalMedia | L110KJ |
| DMEM no D-glucose | BasalMedia | L160KJ |
| DMEM/F12 | BasalMedia | L310KJ |
| DMEM/F12 no D-glucose | Procell | PM150322 |
| RPMI 1640 | BasalMedia | L210KJ |
| β-Mercaptoethanol Solution | Macklin | M917637 |
| 0.25%Trypsin-EDTA(1X) | NCM Biotech | C100C1 |
| Penicillin-Streptomycin | NCM Biotech | C100C5 |
| RIPA Lysis Buffer | NCM Biotech | WB3100 |
| Skim Milk | YEASEN | 36120ES76 |
| Tris-MOPS-SDS Runing Buffer Powder | GenScript | M00138 |
| 10X TBST Buffer | Sangon Biotech | C520009-0001 |
| Antibody Diluent | NCM Biotech | WB500D |
| NcmECL Ultra | NCM Biotech | P10300 |
| 4% Paraformaldehyde | Biosharp | BL539A |
| DAPI Staining Solution | Beyotime | C1005 |
| Microplate | Thermo Fisher | 4346906 |

| **Software and algorithms** | **Source** | **Identifier** |
| --- | --- | --- |
| GEPIA2 | Tang, et al^1^. | http://gepia2.cancer-pku.cn/#index |
| LinkedOmicsKB | Liao, et al^2^. | https://kb.linkedomics.org/ |
| GraphPad Prism 9.4.0 | GraphPad Software | https://www.graphpad.com/scientific-software/prism/ |
| R version 4.1.1 | R Core Team | https://www.r-project.org |
| Biorender | NA | https://biorender.com/ |
| TIMER 2.0 | Li, et al^3^. | http://timer.cistrome.org/ |
| Image J | National Institutes of Health | https://imagej.nih.gov/ij/ |

1 Tang, Z., Kang, B., Li, C., Chen, T. & Zhang, Z. GEPIA2: an enhanced web server for large-scale expression profiling and interactive analysis. *Nucleic Acids Res* **47**, W556-W560, doi:10.1093/nar/gkz430 (2019).

2 Liao, Y. *et al.* A proteogenomics data-driven knowledge base of human cancer. *Cell Syst* **14**, 777-787 e775, doi:10.1016/j.cels.2023.07.007 (2023).

3 Li, T. *et al.* TIMER2.0 for analysis of tumor-infiltrating immune cells. *Nucleic Acids Res* **48**, W509-W514, doi:10.1093/nar/gkaa407 (2020).
