# Supplementary material for: Targeting CXCL8 signaling sensitizes HNSCC to anlotinib by reducing tumor-associated macrophage-derived CLU
Source: J Exp Clin Cancer Res. 2025 Feb 5;44:39. doi: 10.1186/s13046-025-03298-7 (PMC11796229; doi:10.1186/s13046-025-03298-7)

Supplementary Figure 1

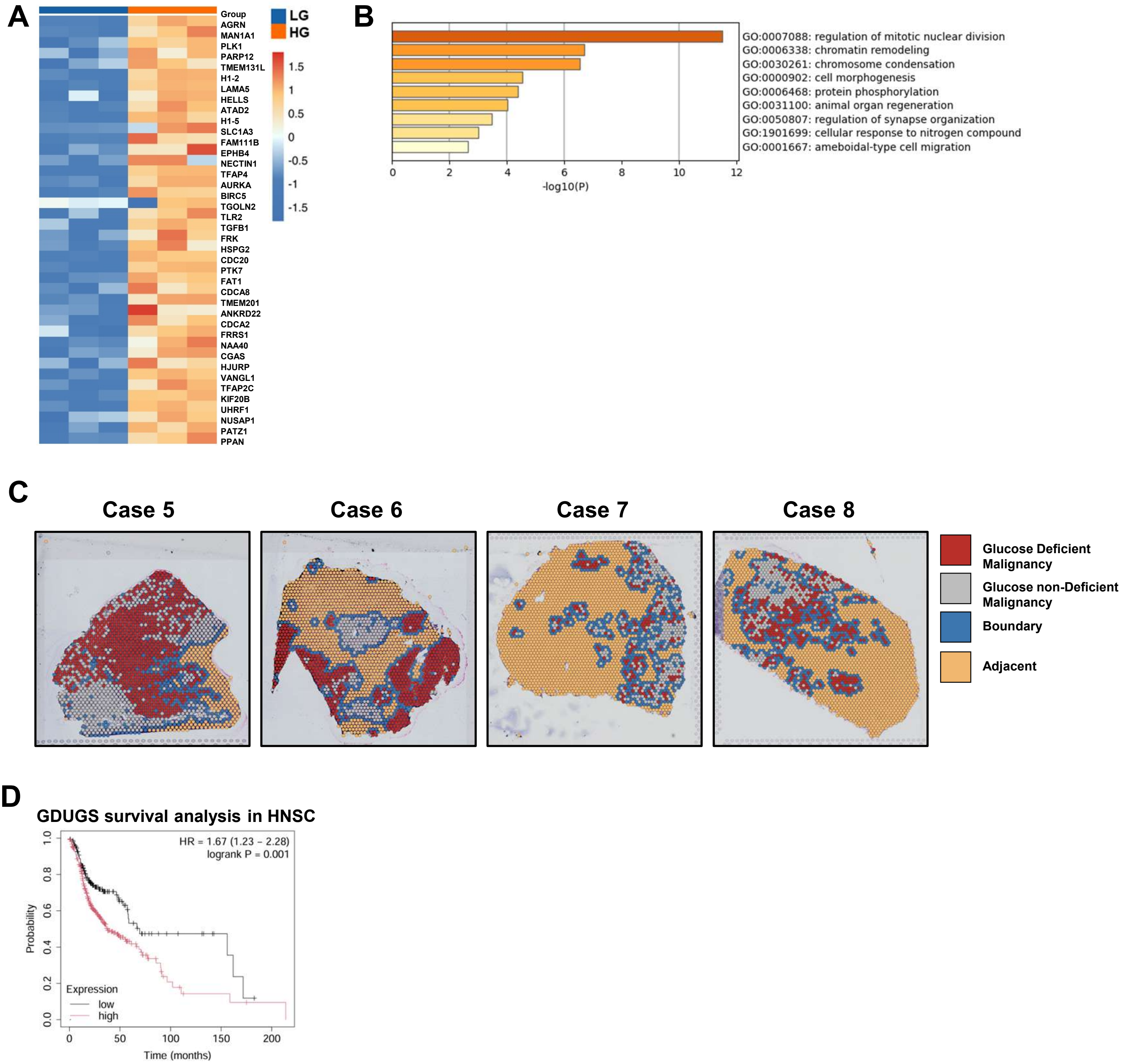

Supplementary Figure 2

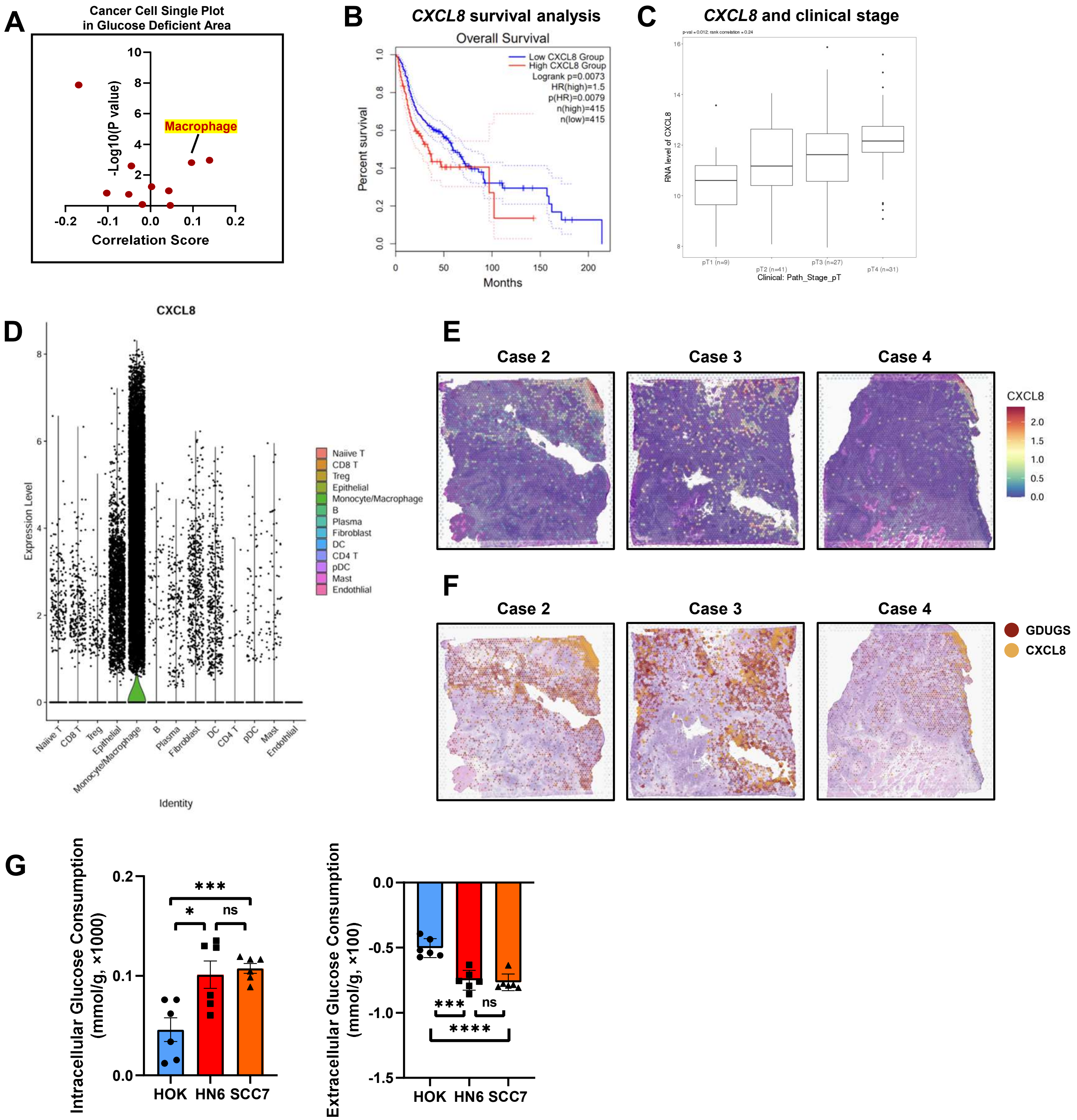

Supplementary Figure 3

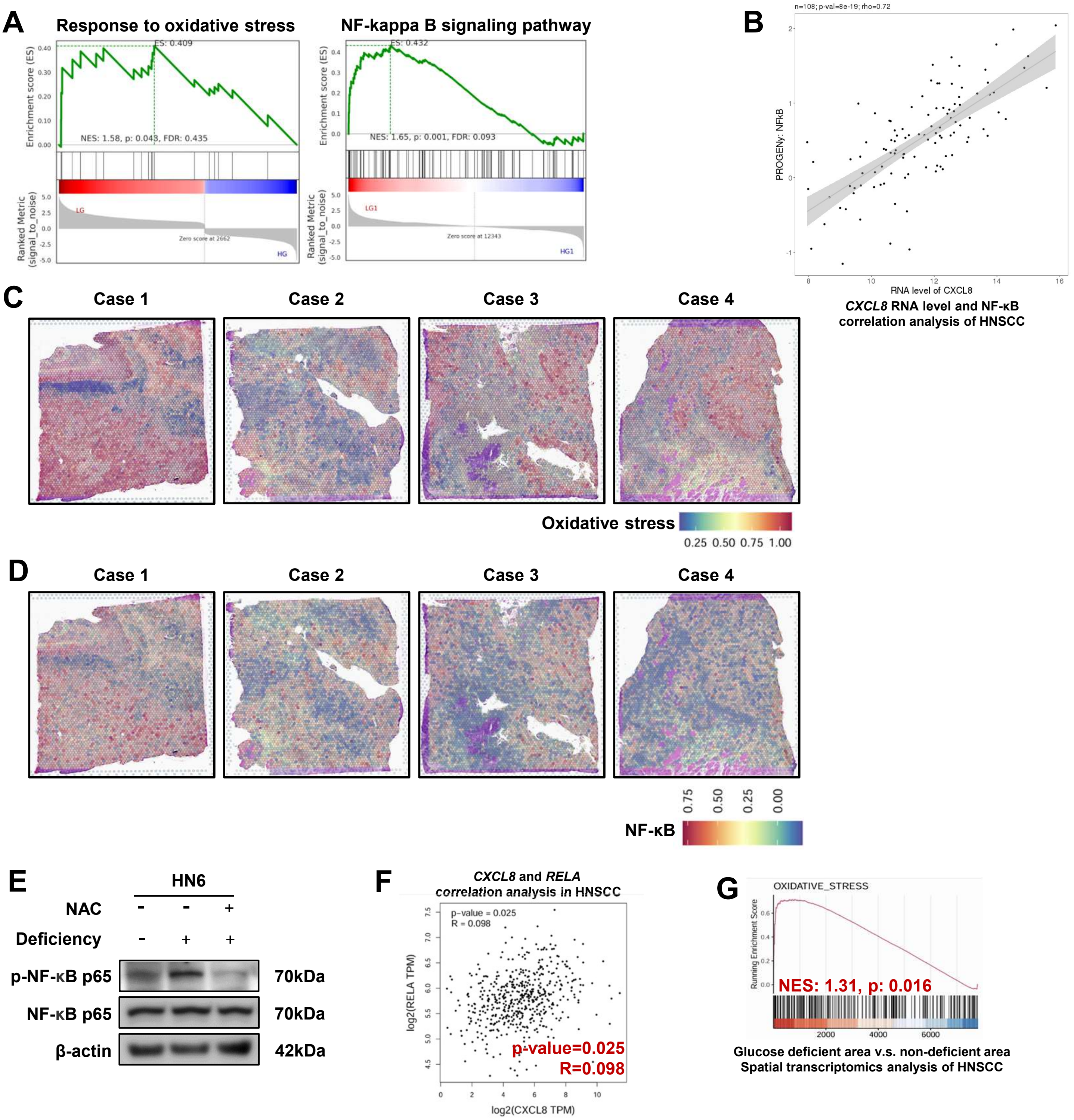

Supplementary Figure 4

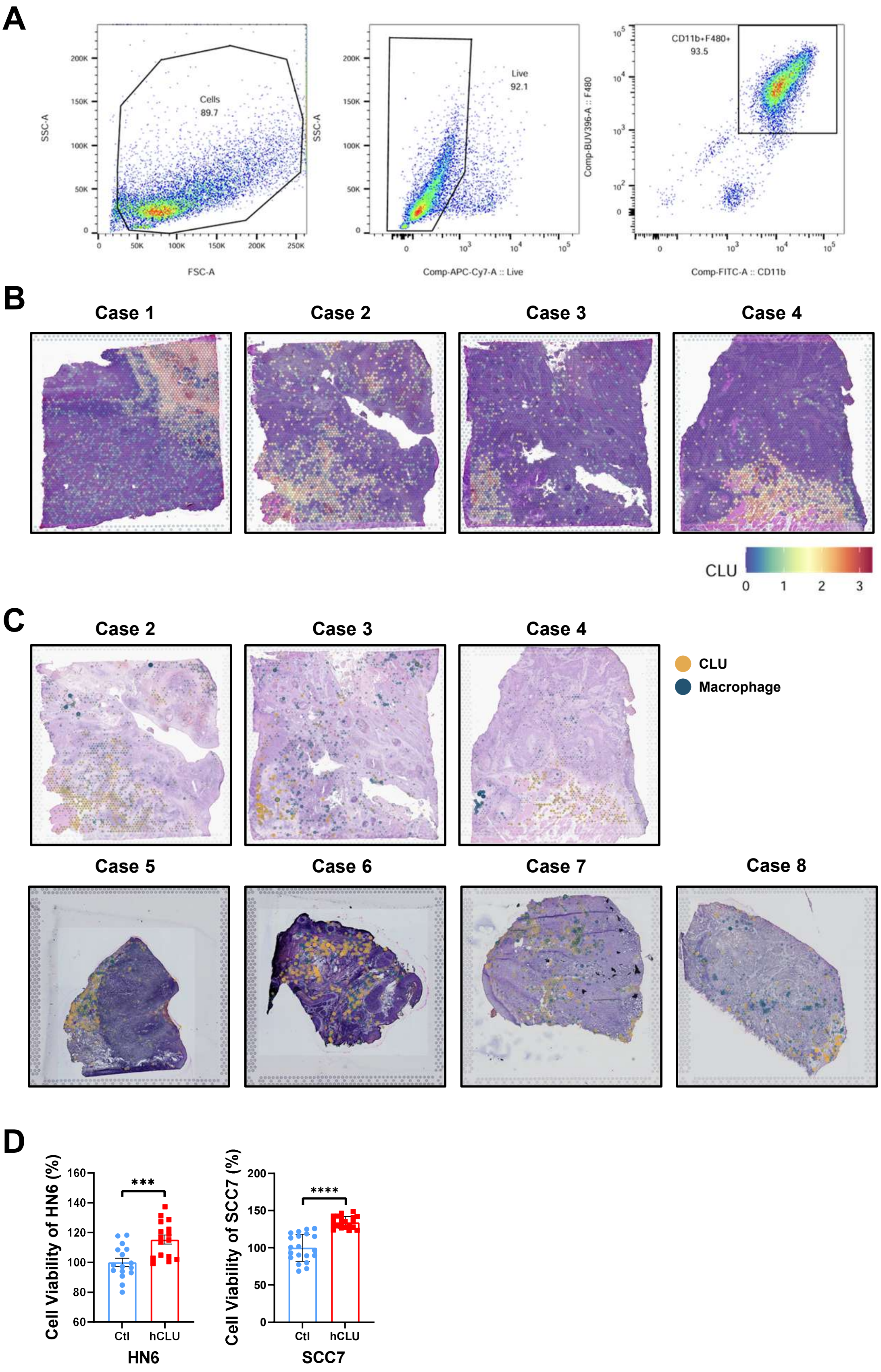

Supplementary Figure 5

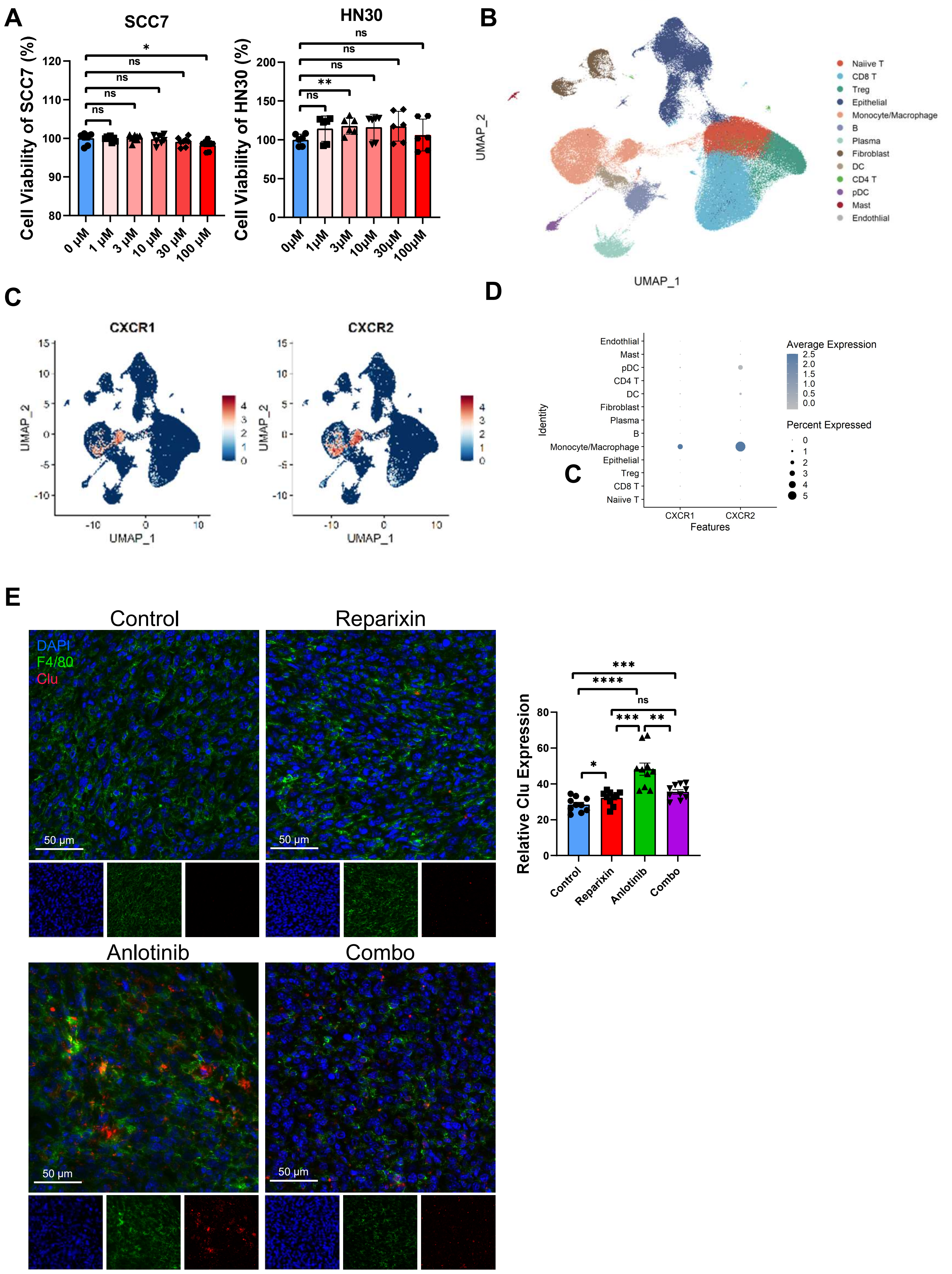

Supplementary Figure 6

A

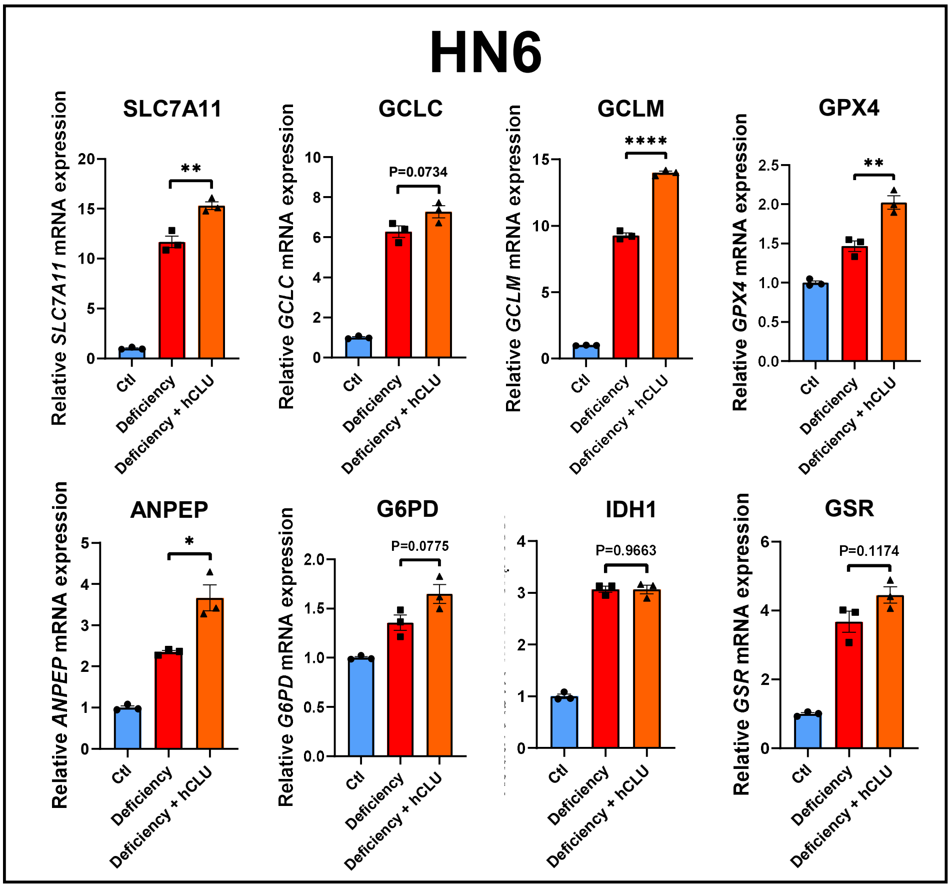

B

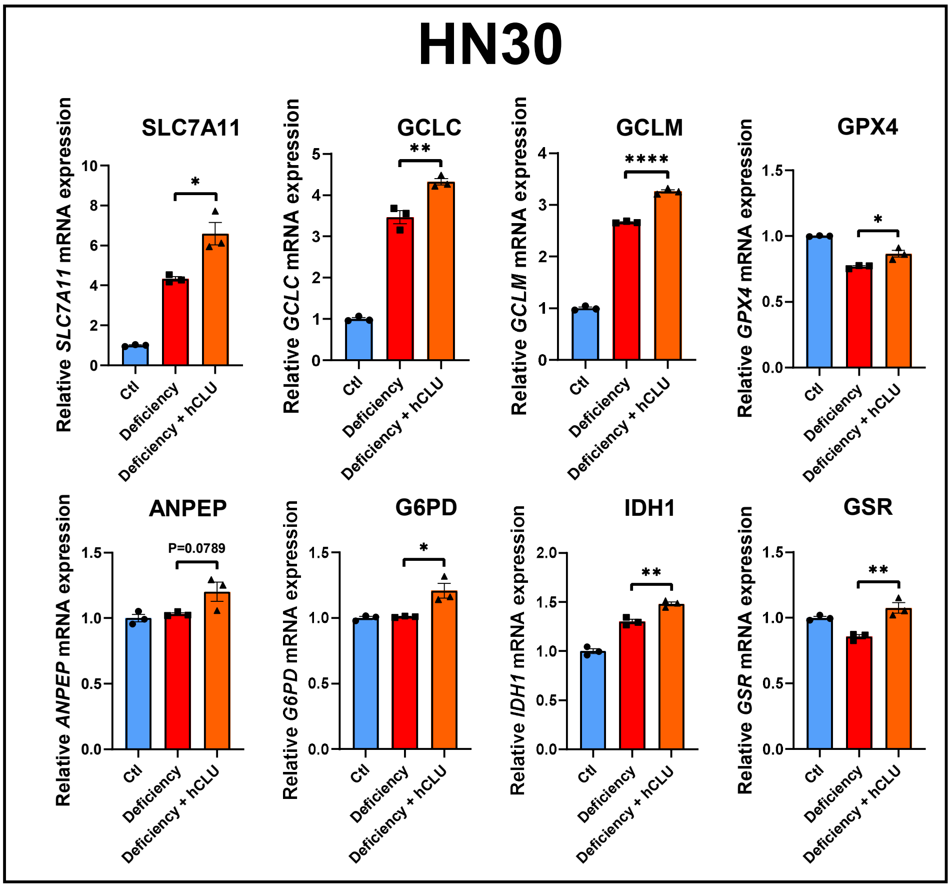

Supplement: Supplementary file 4 — Supplementary Material 4. [file 13046_2025_3298_MOESM4_ESM.pdf]
